# Supplementary material for: The calmodulin redox sensor controls myogenesis
Source: PLoS One. 2020 Sep 17;15(9):e0239047. doi: 10.1371/journal.pone.0239047 (PMC7498019; doi:10.1371/journal.pone.0239047)
Supplement: S1 Raw images — (PDF) [file pone.0239047.s001.pdf]

Western blot in Figure 3C

Probed with anti-MyoD and anti-biotin (ladder):

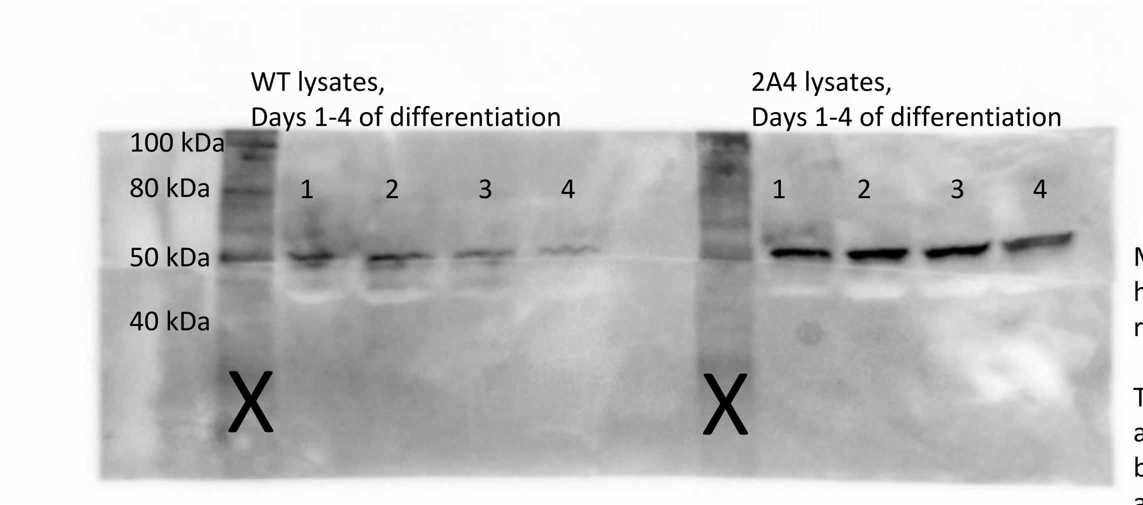

MyoD is 34kDa, but is heavily glycosylated and runs at 50 kDa

This blot was stripped and re-probed after blotting with anti pan-actin, so it had been cut and reassembled along the 50 kDa region.

Probed with anti-pan actin & anti-biotin (ladder):

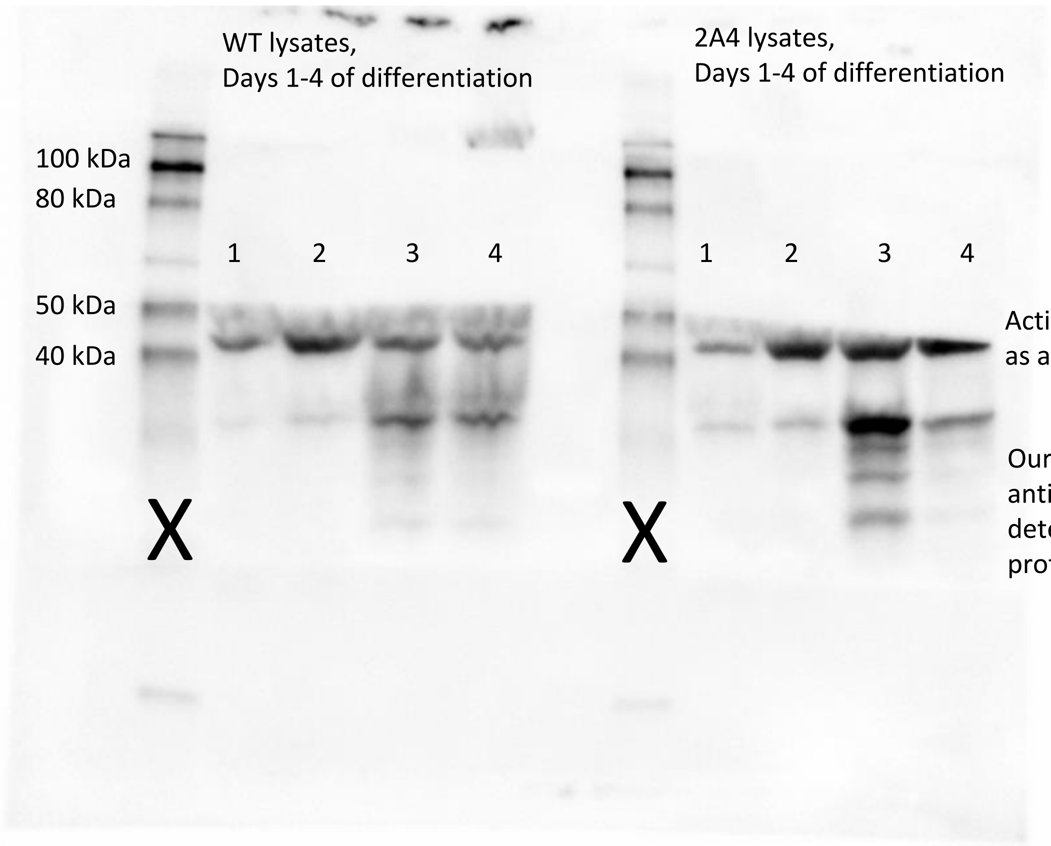

Actin is 45kDa--we used this band as a loading control

Our polyclonal pan-actin antibody non-specifically detected some small MW proteins.
