# Supplementary material for: The calmodulin redox sensor controls myogenesis
Source: PLoS One. 2020 Sep 17;15(9):e0239047. doi: 10.1371/journal.pone.0239047 (PMC7498019; doi:10.1371/journal.pone.0239047)

Representative immmunofluorescence images acquired for myogenic factors and muscle proteins on days 0-4 of differentiation for wild type (WT) and *CALM1* M109Q (2A4) C2C12 cells

Day 0 p-p53

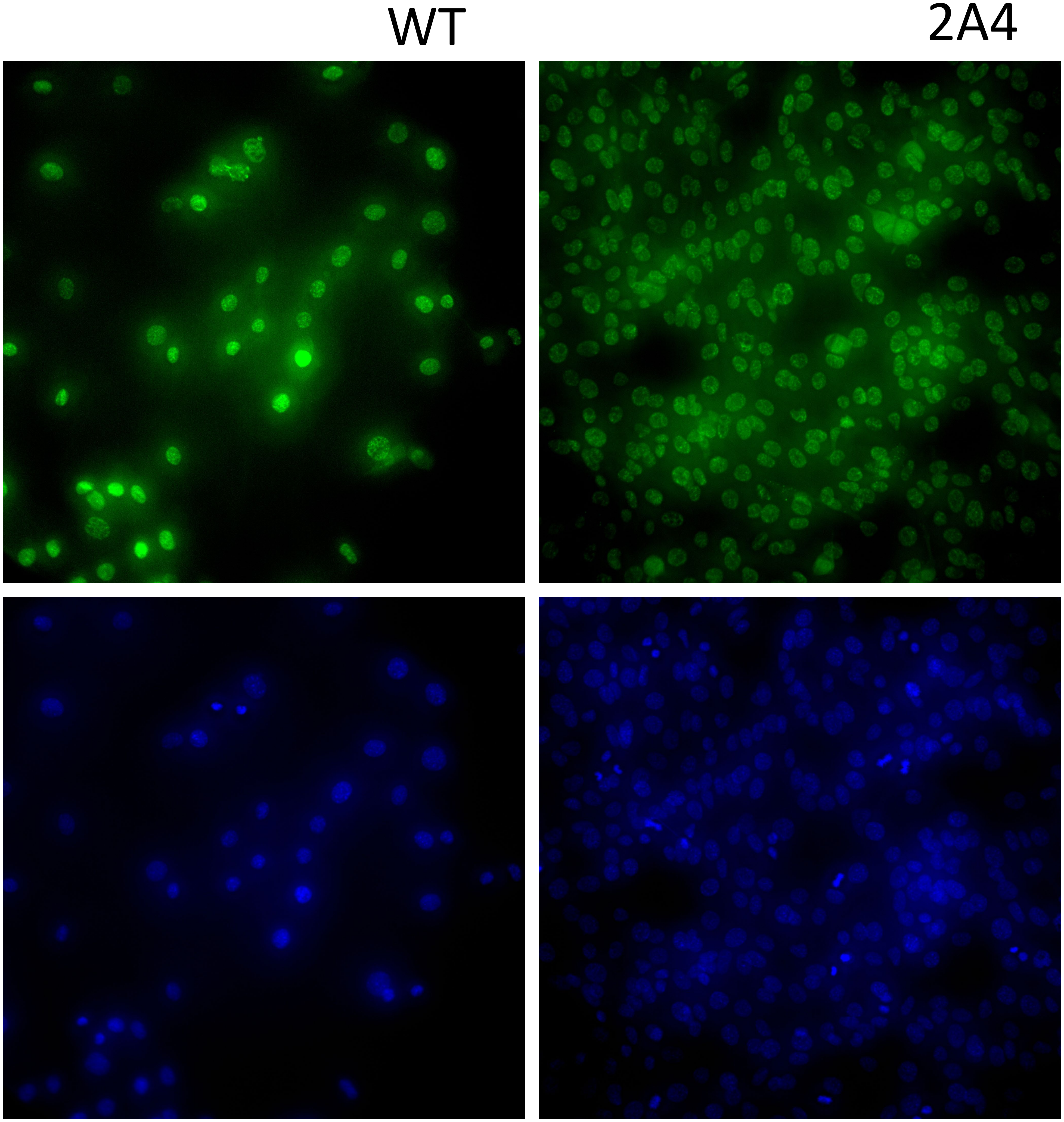

Day 1 p-p53

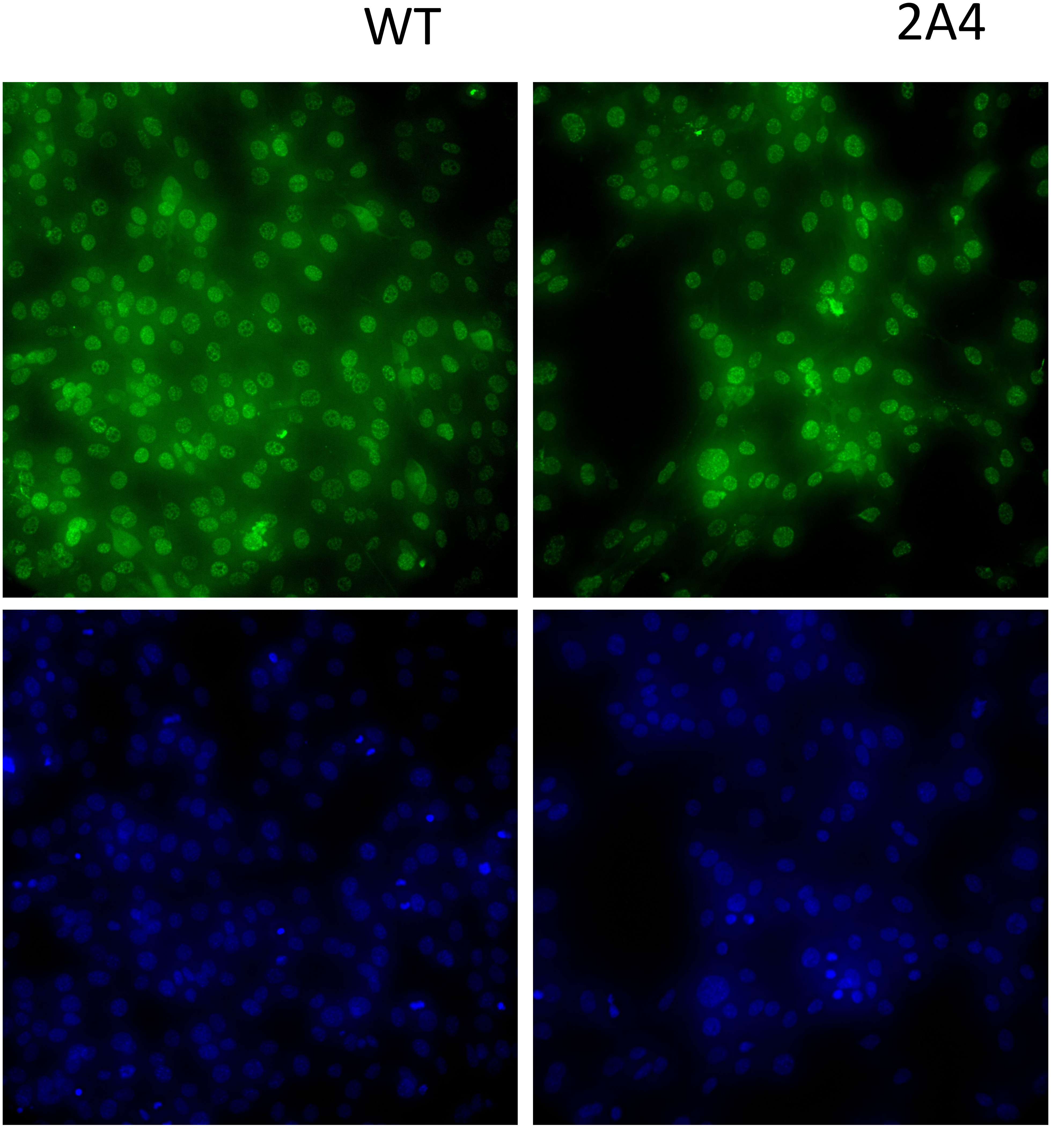

Day 2 p-p53

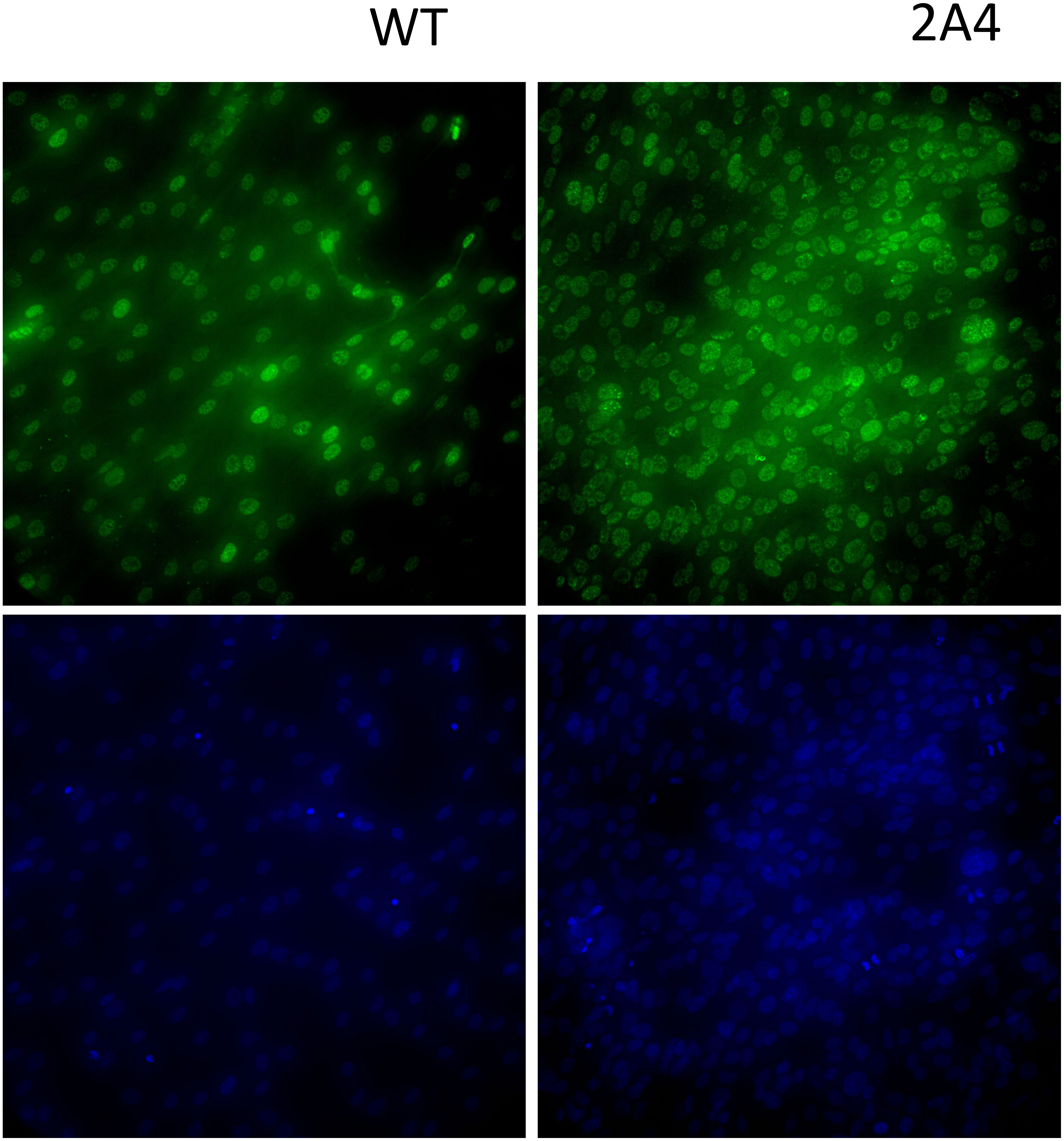

Day 3 p-p53

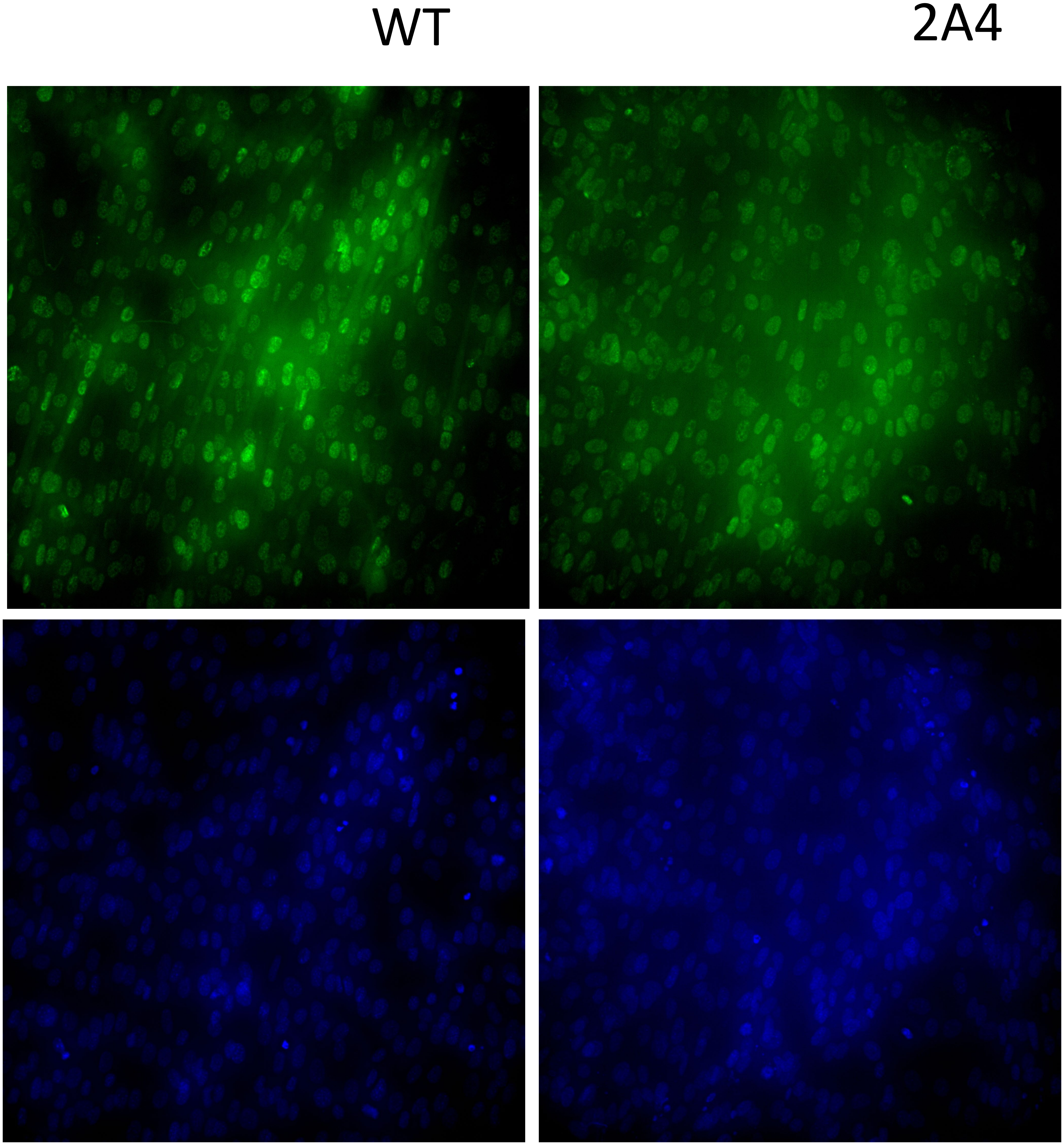

Day 4 p-p53

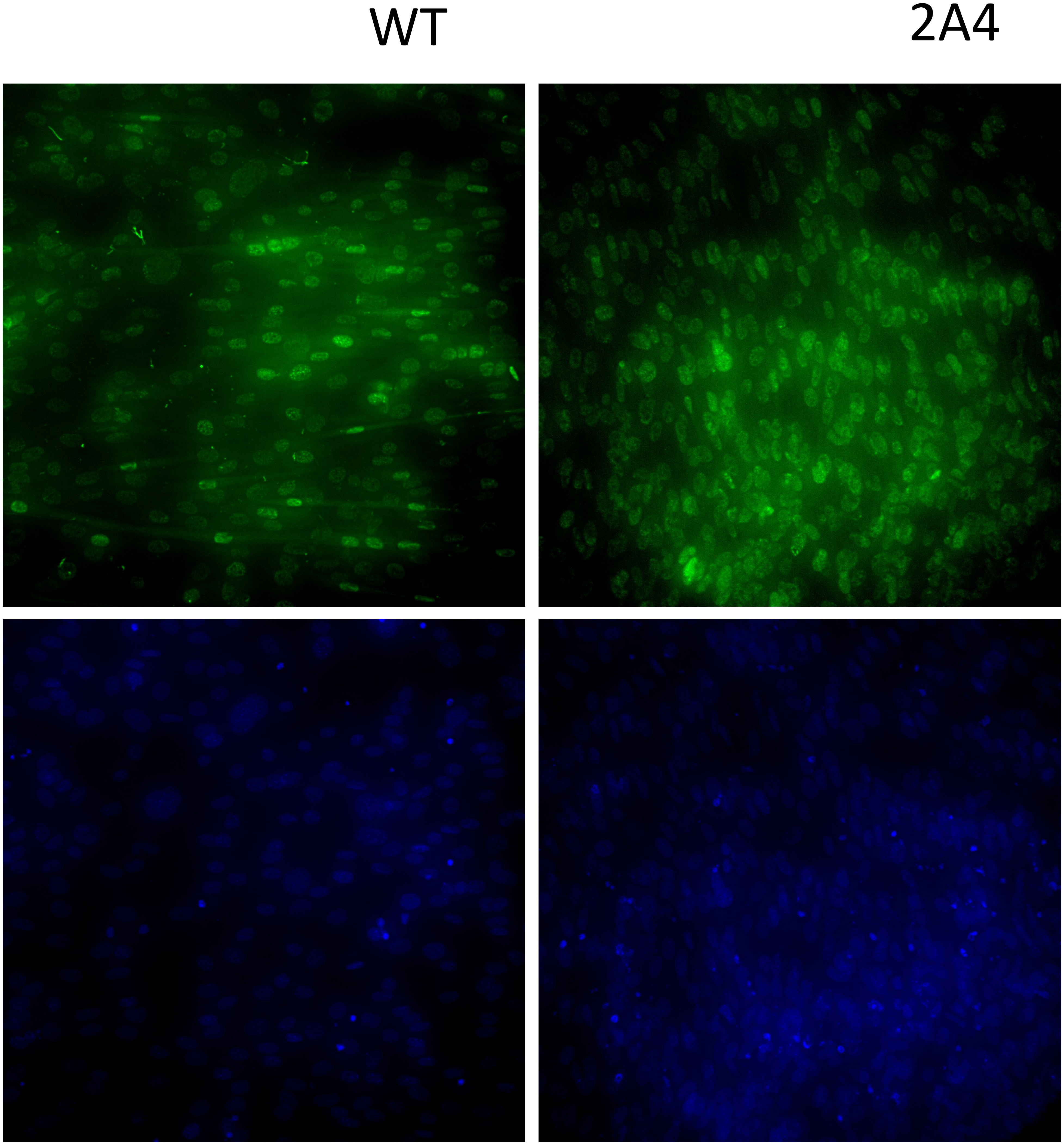

Day 0 p53

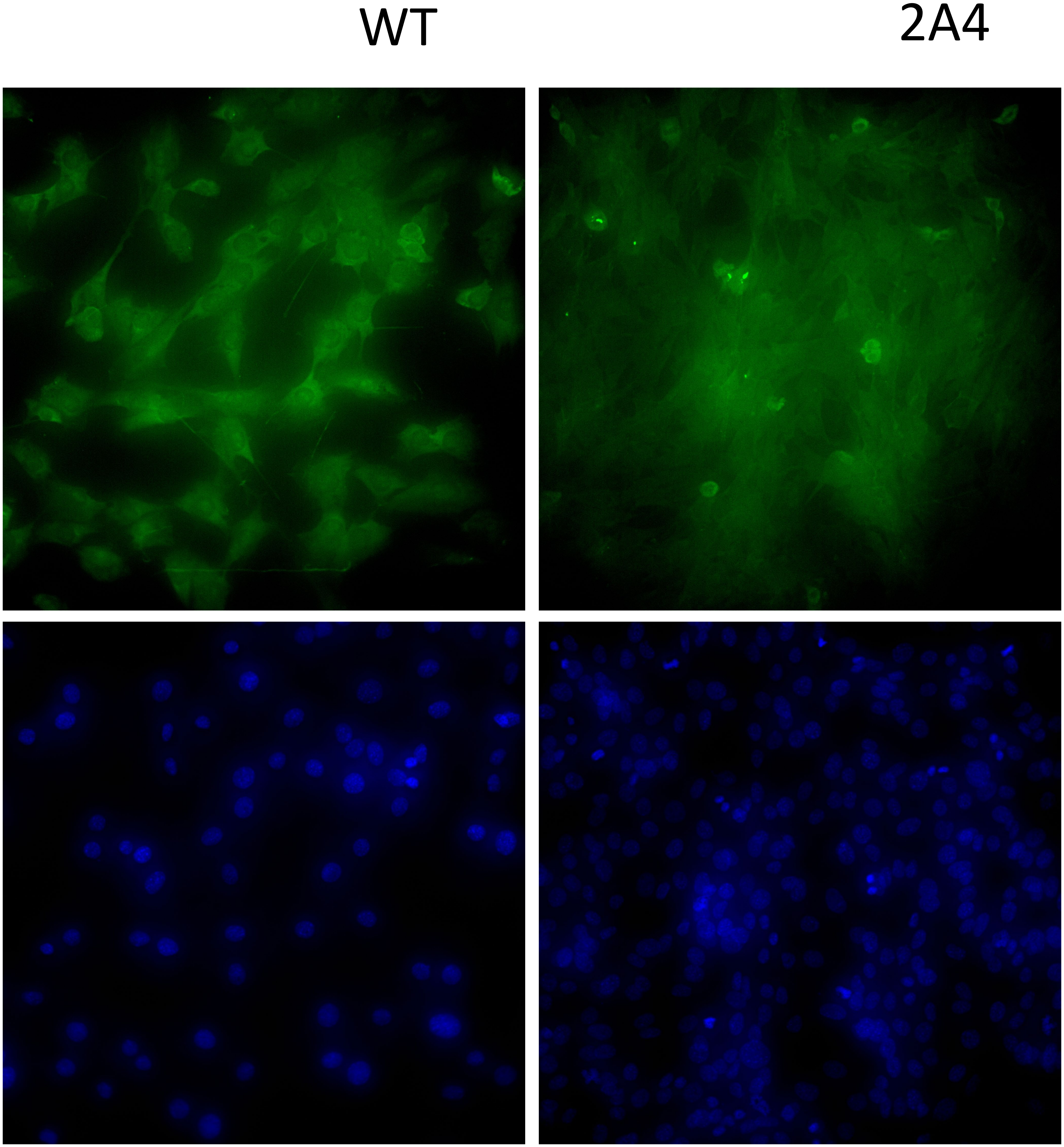

Day 1 p53

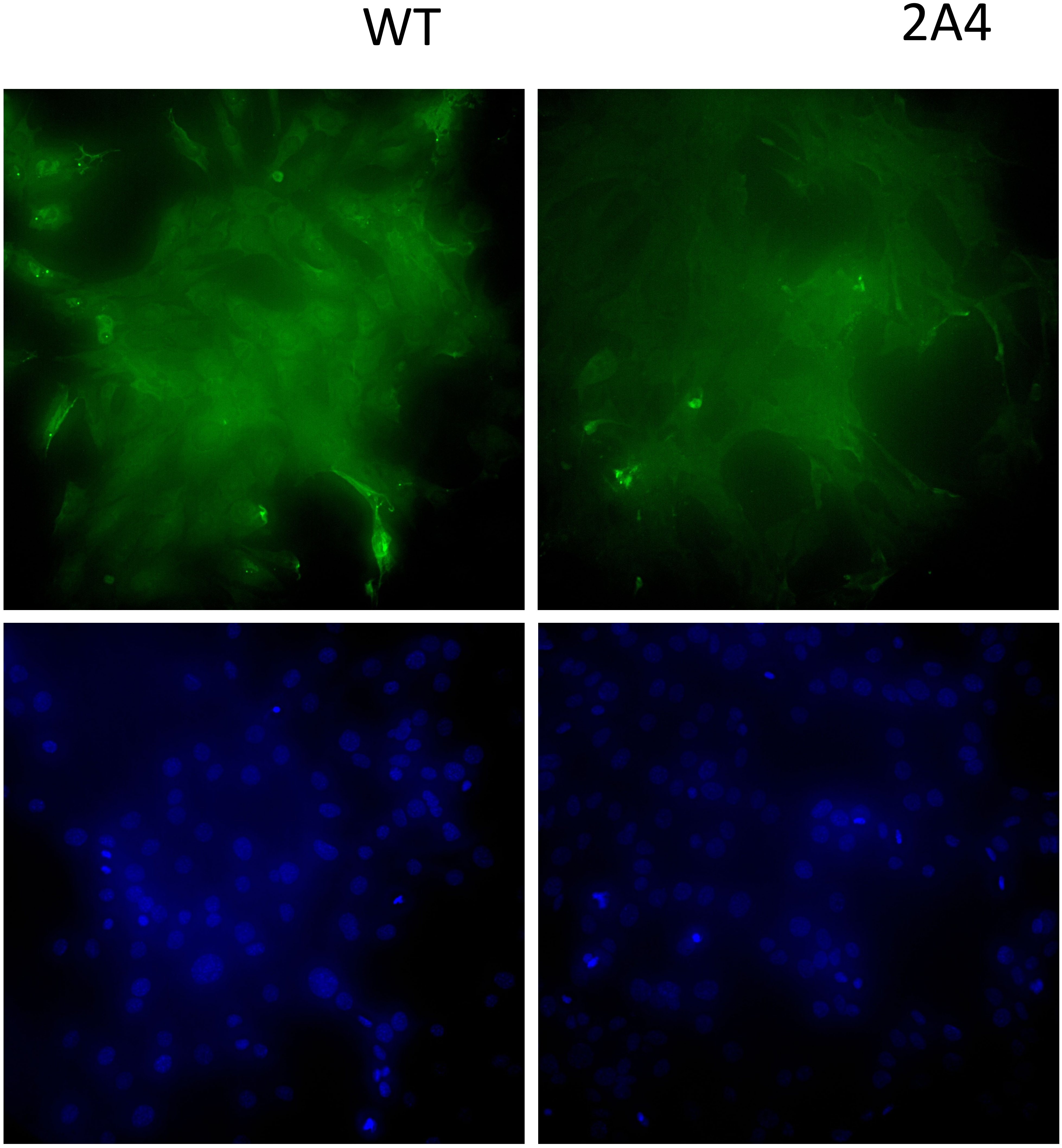

Day 2 p53

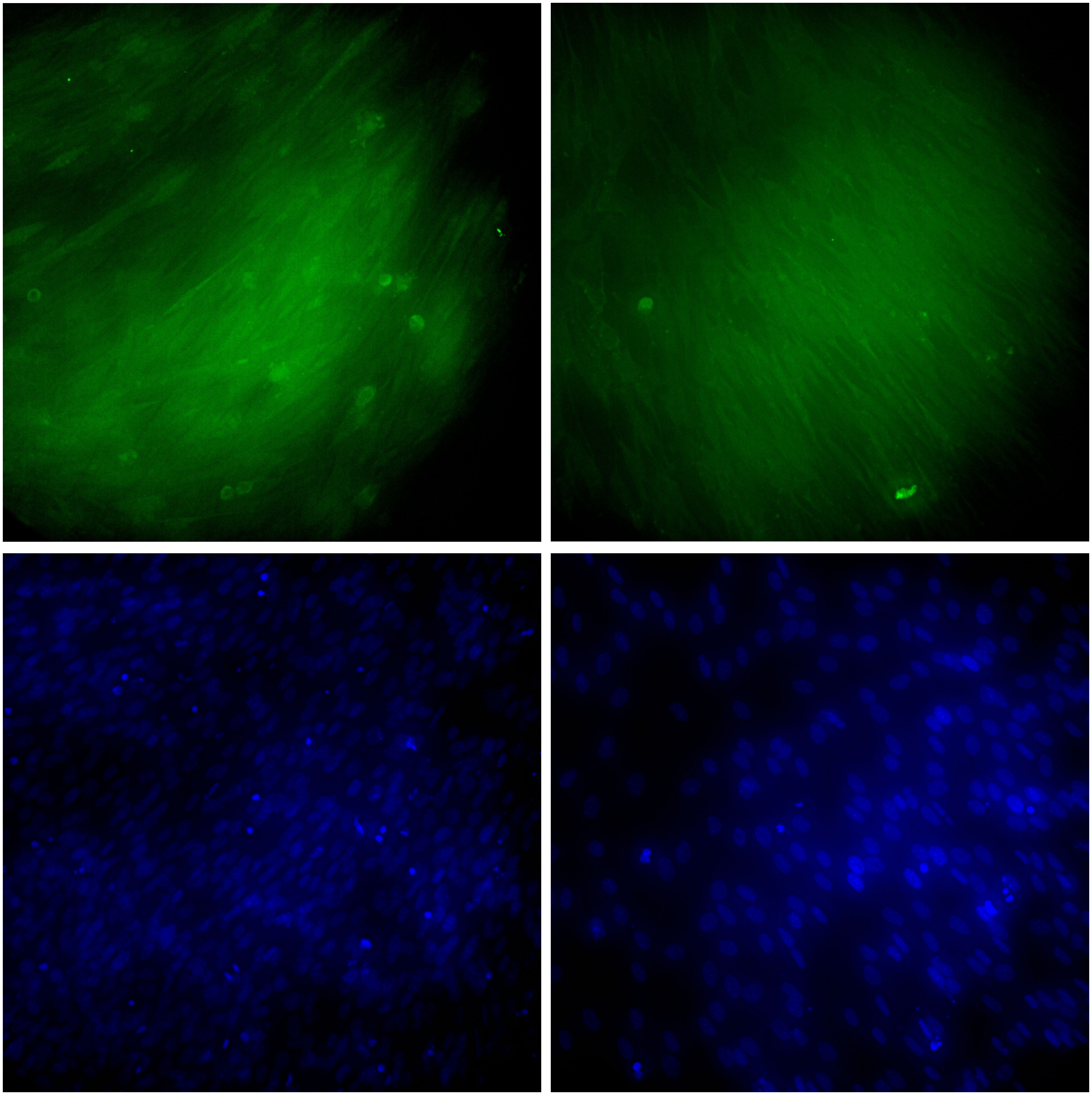

Day 3 p53

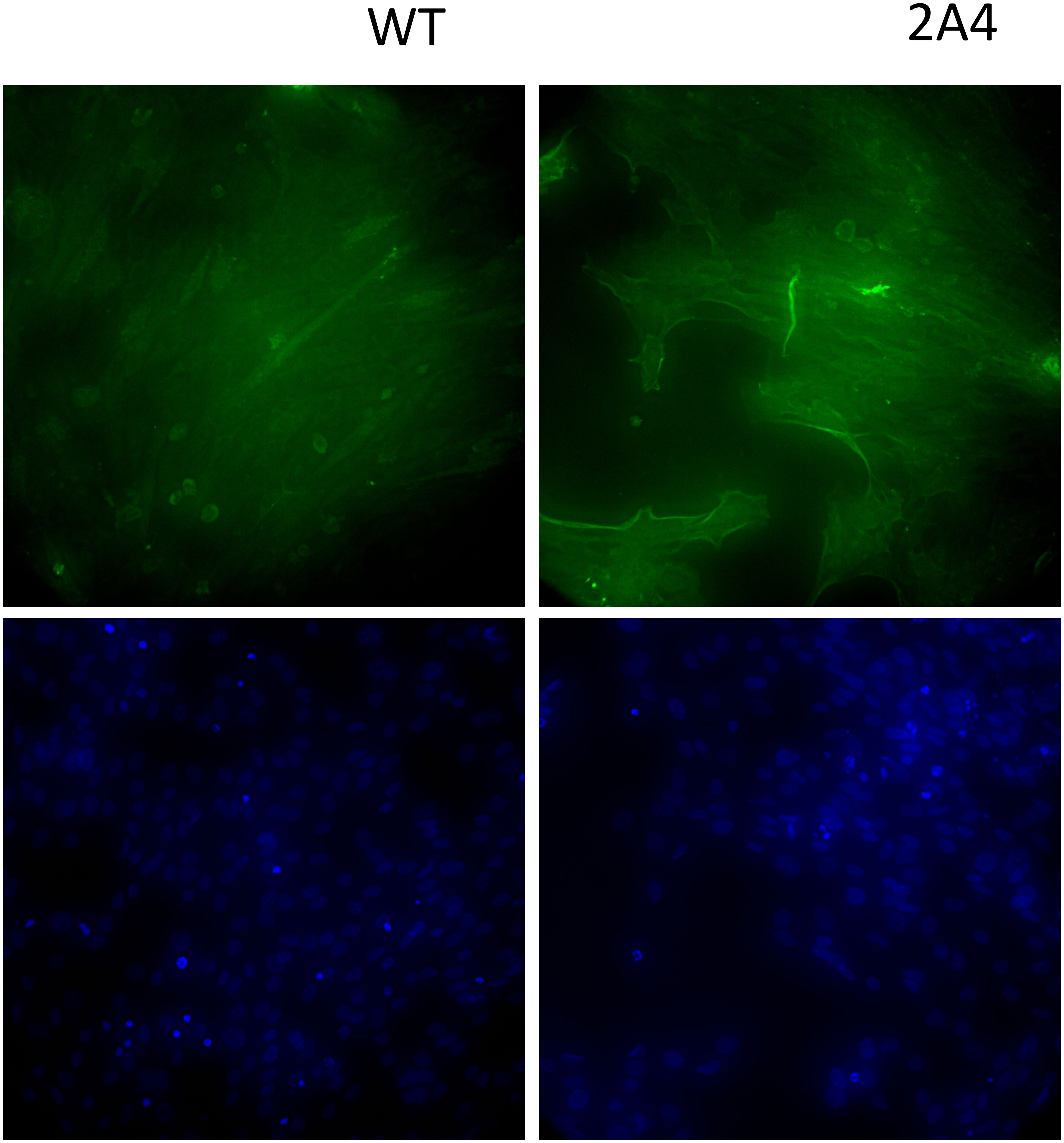

Day 4 p53

WT

2A4

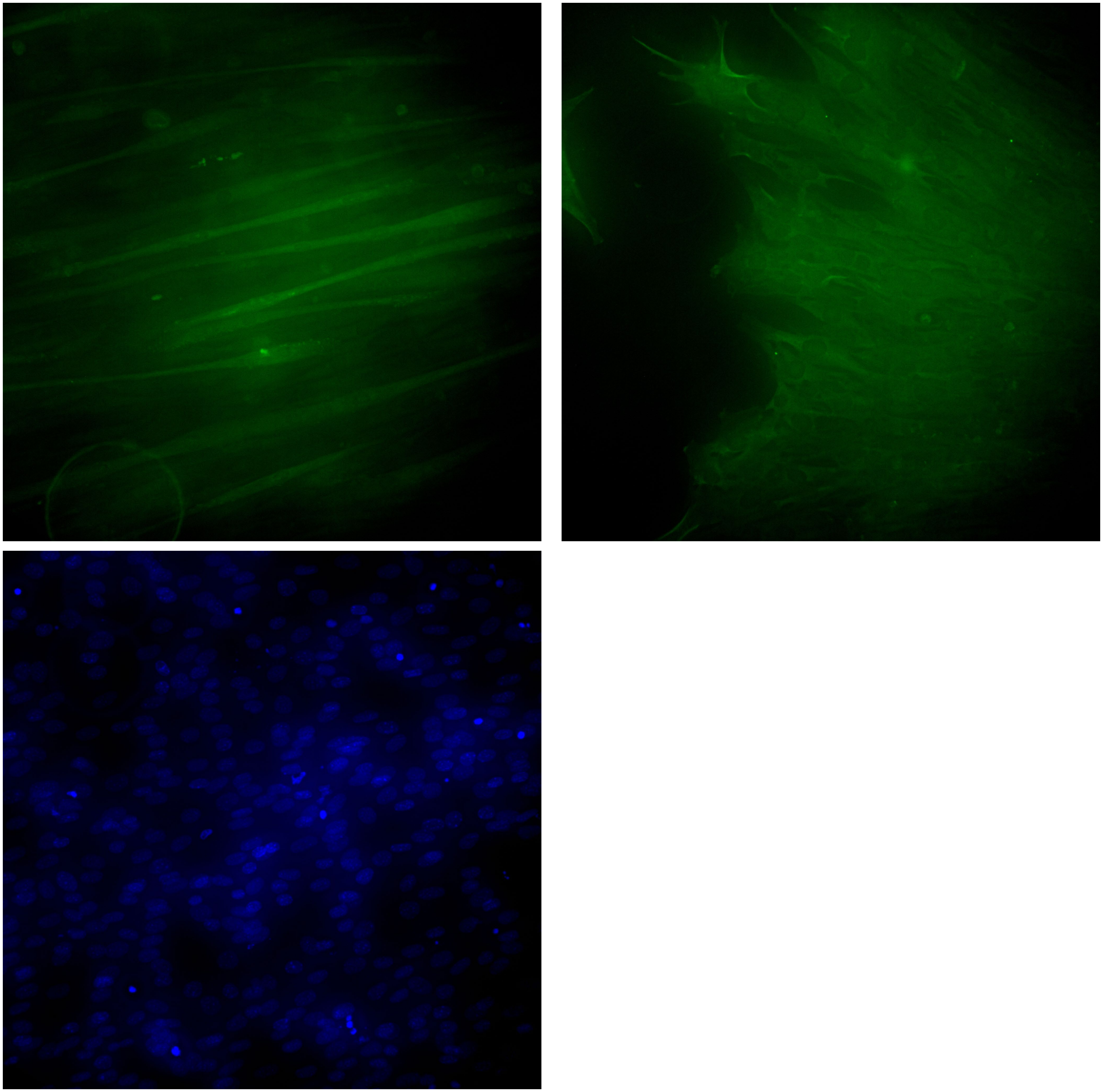

Day 0 p21

WT 2A4

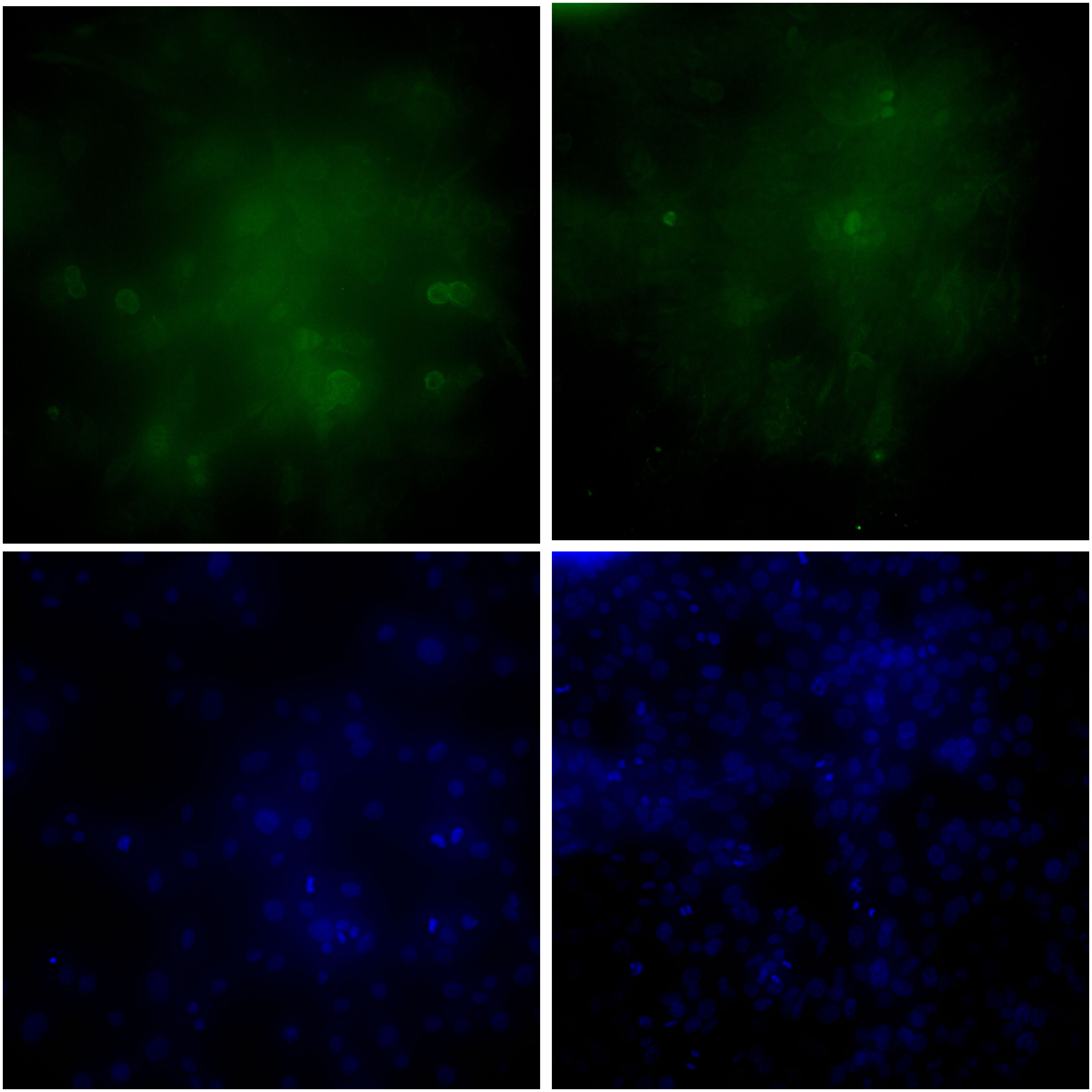

Day 1 p21

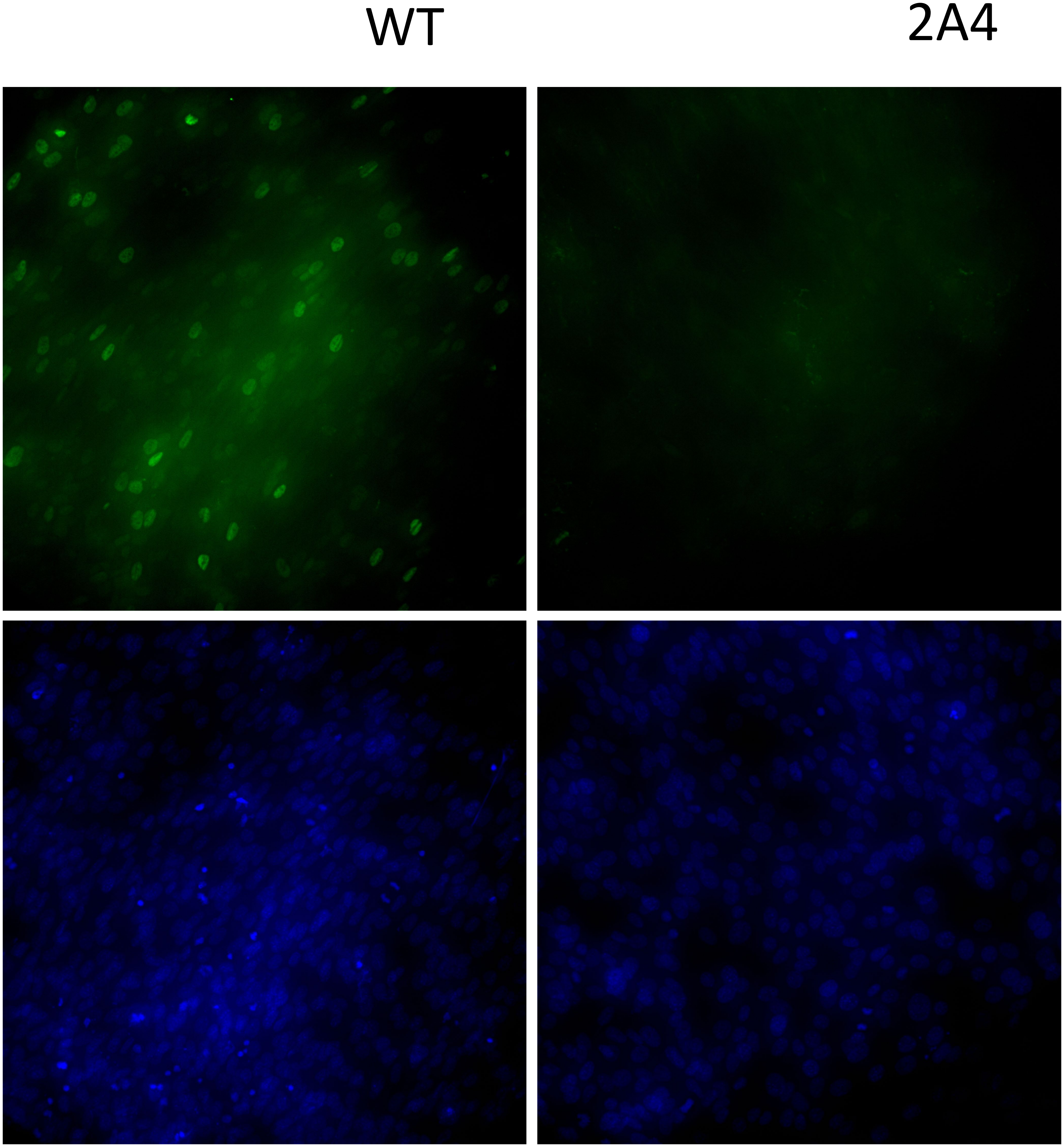

Day 2 p21

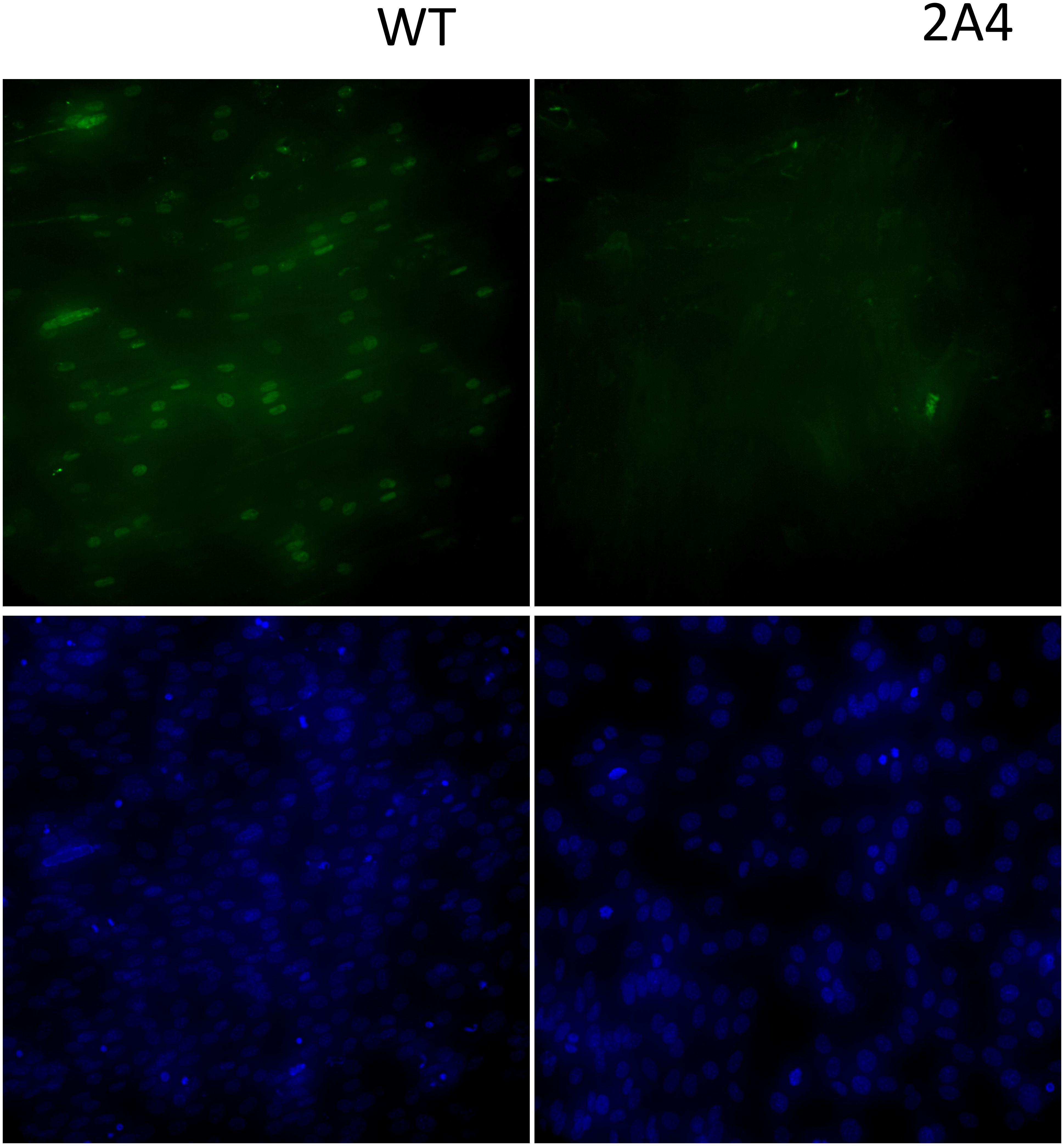

Day 3 p21

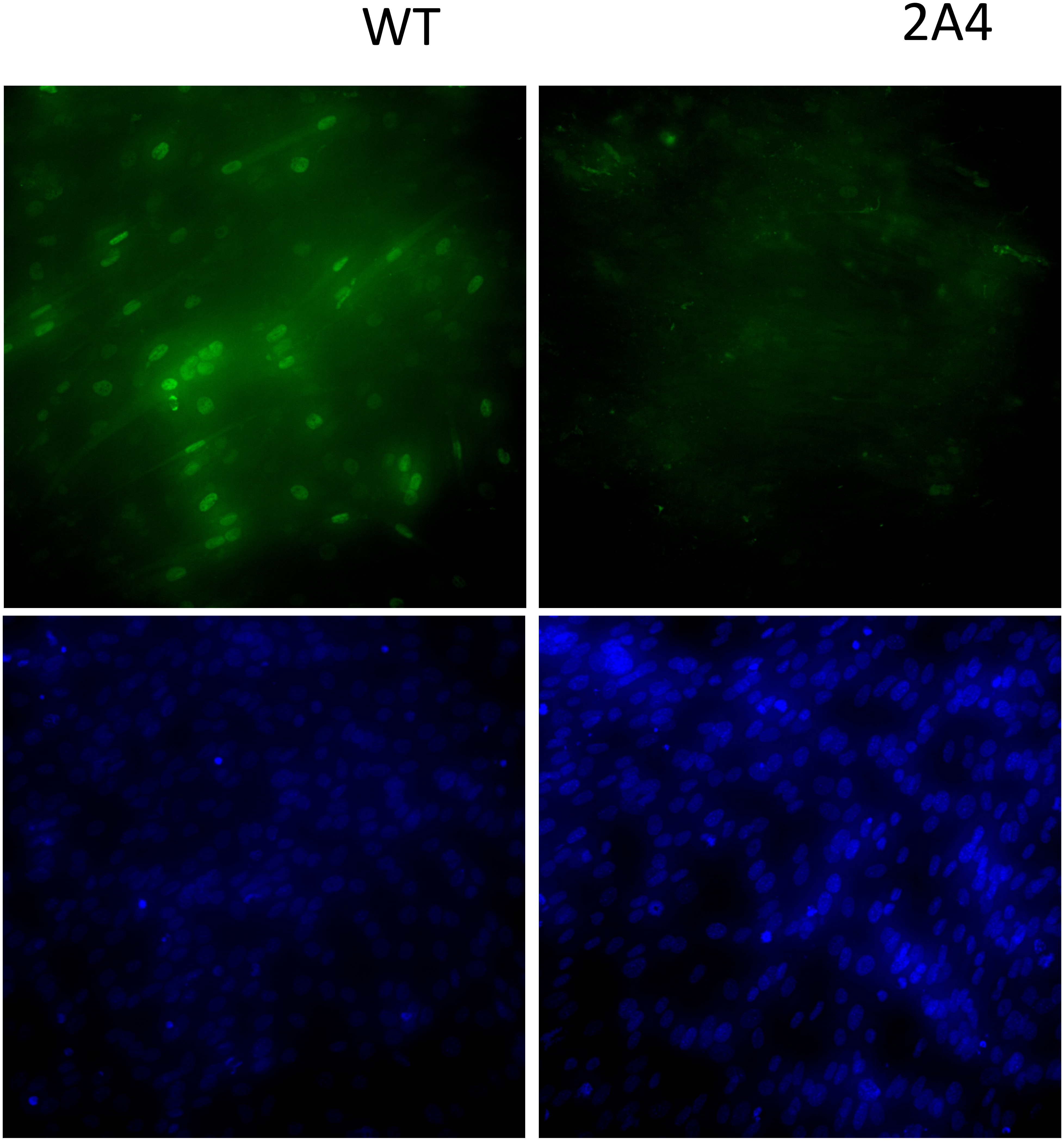

Day 4 p21

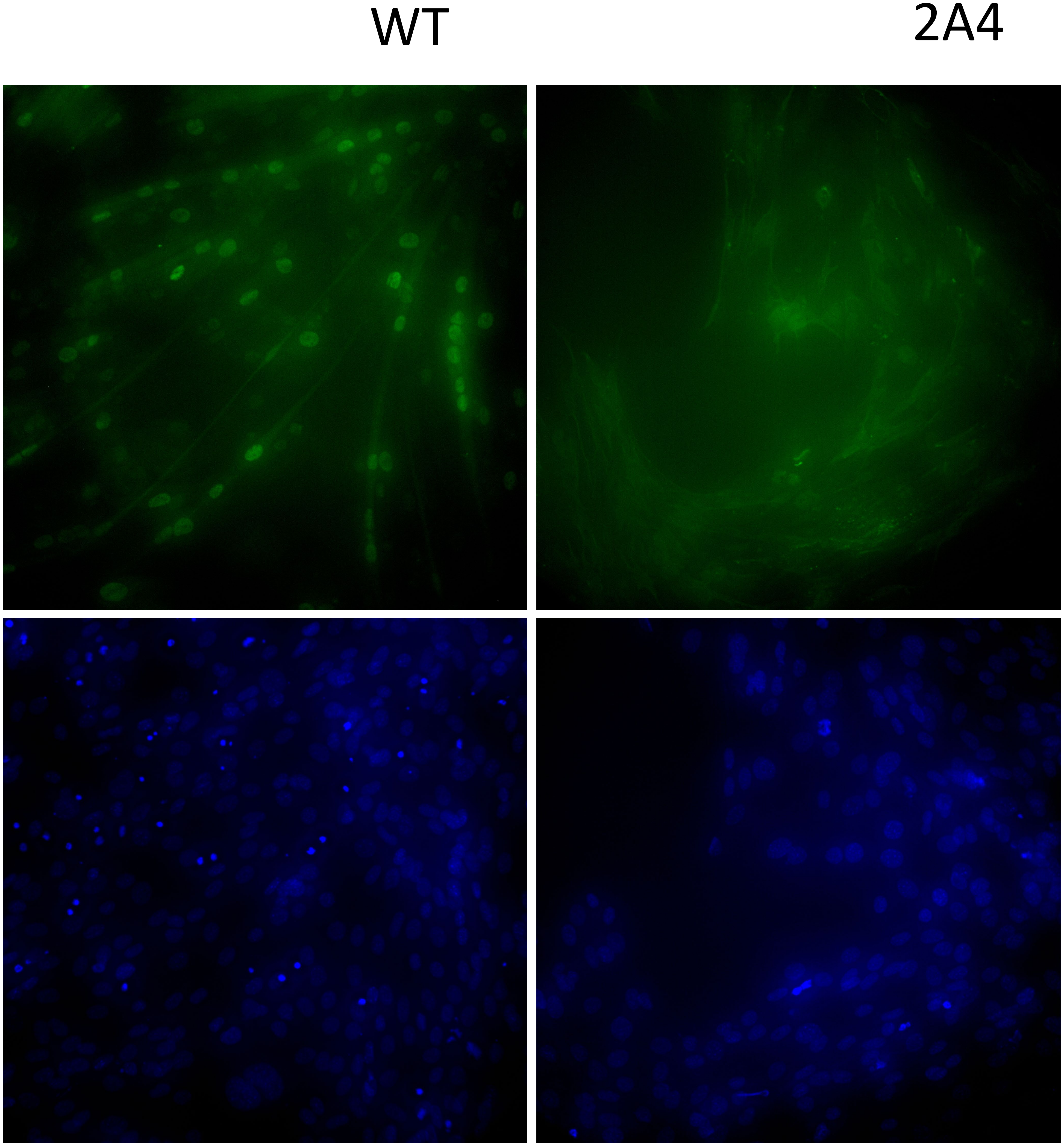

Day 0 MyoD

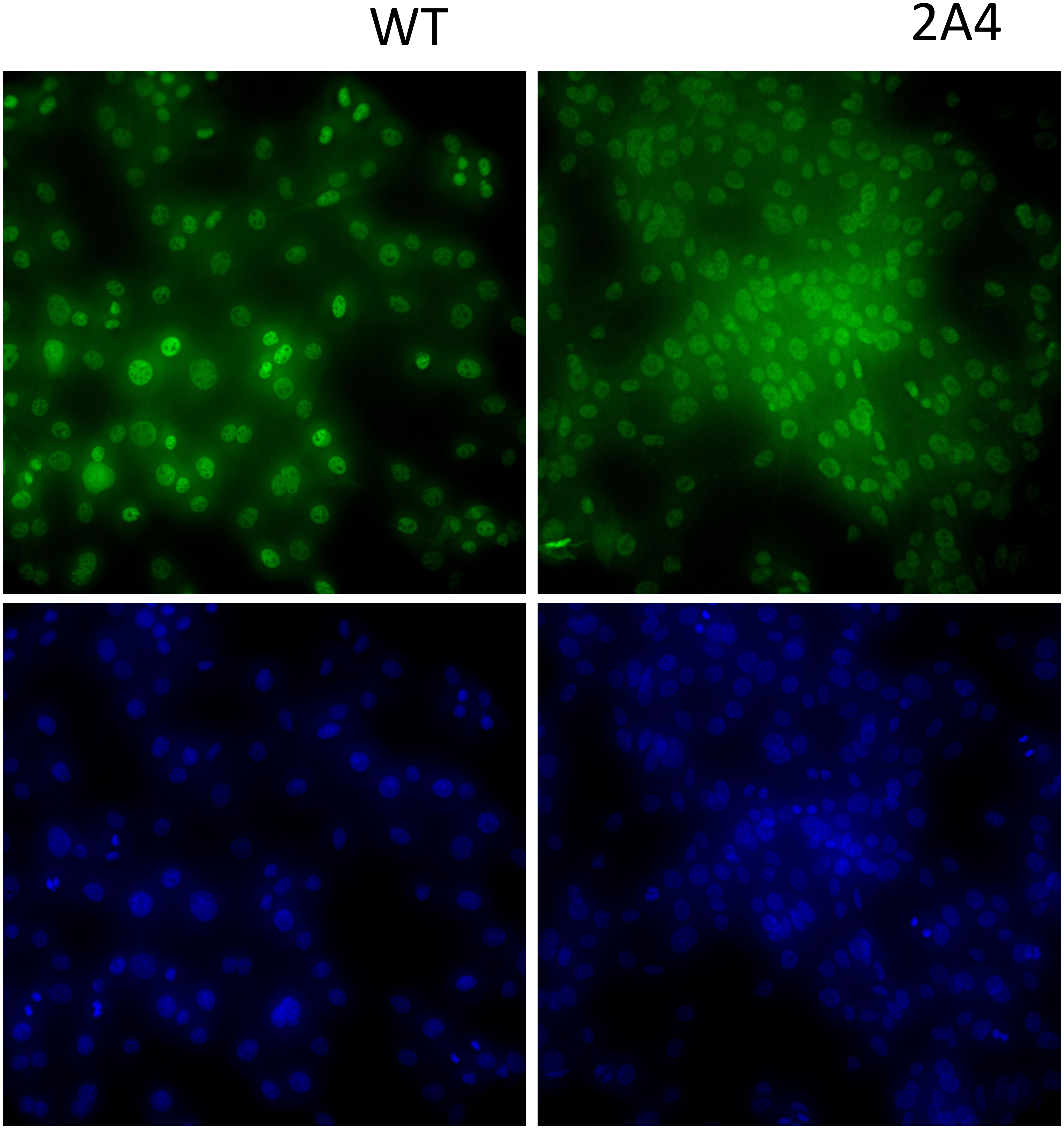

Day 1 MyoD

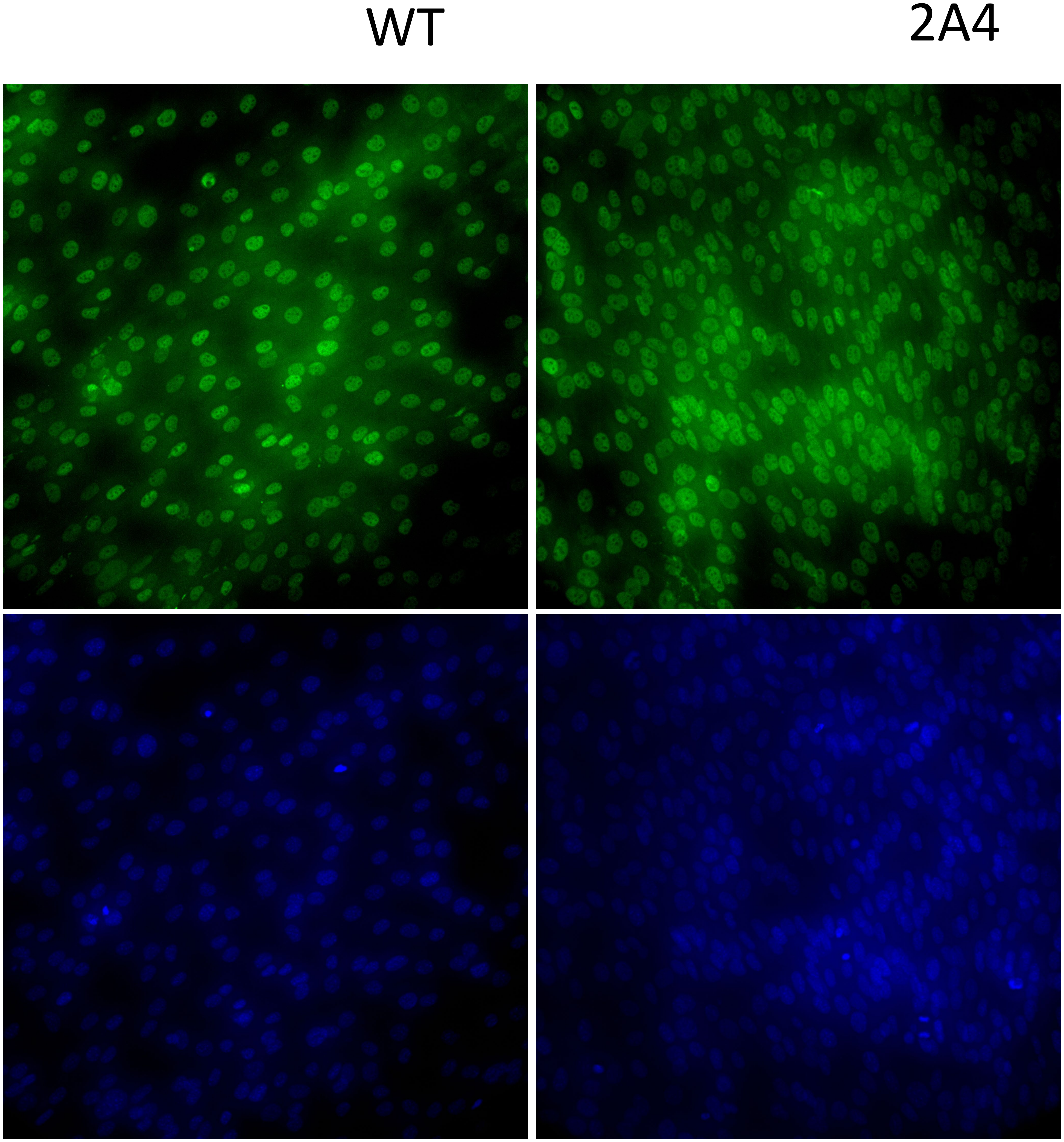

Day 2 MyoD

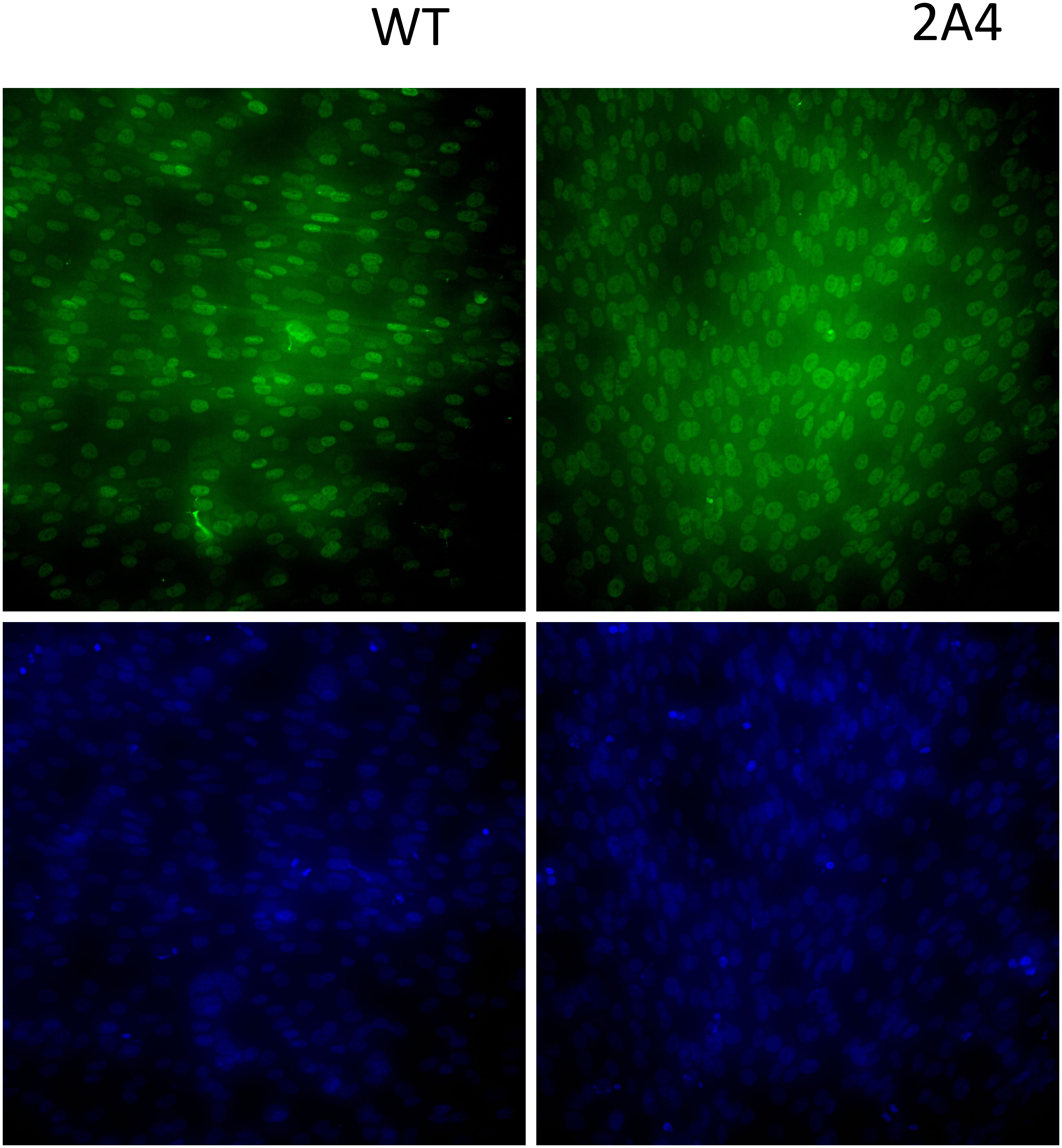

Day 3 MyoD

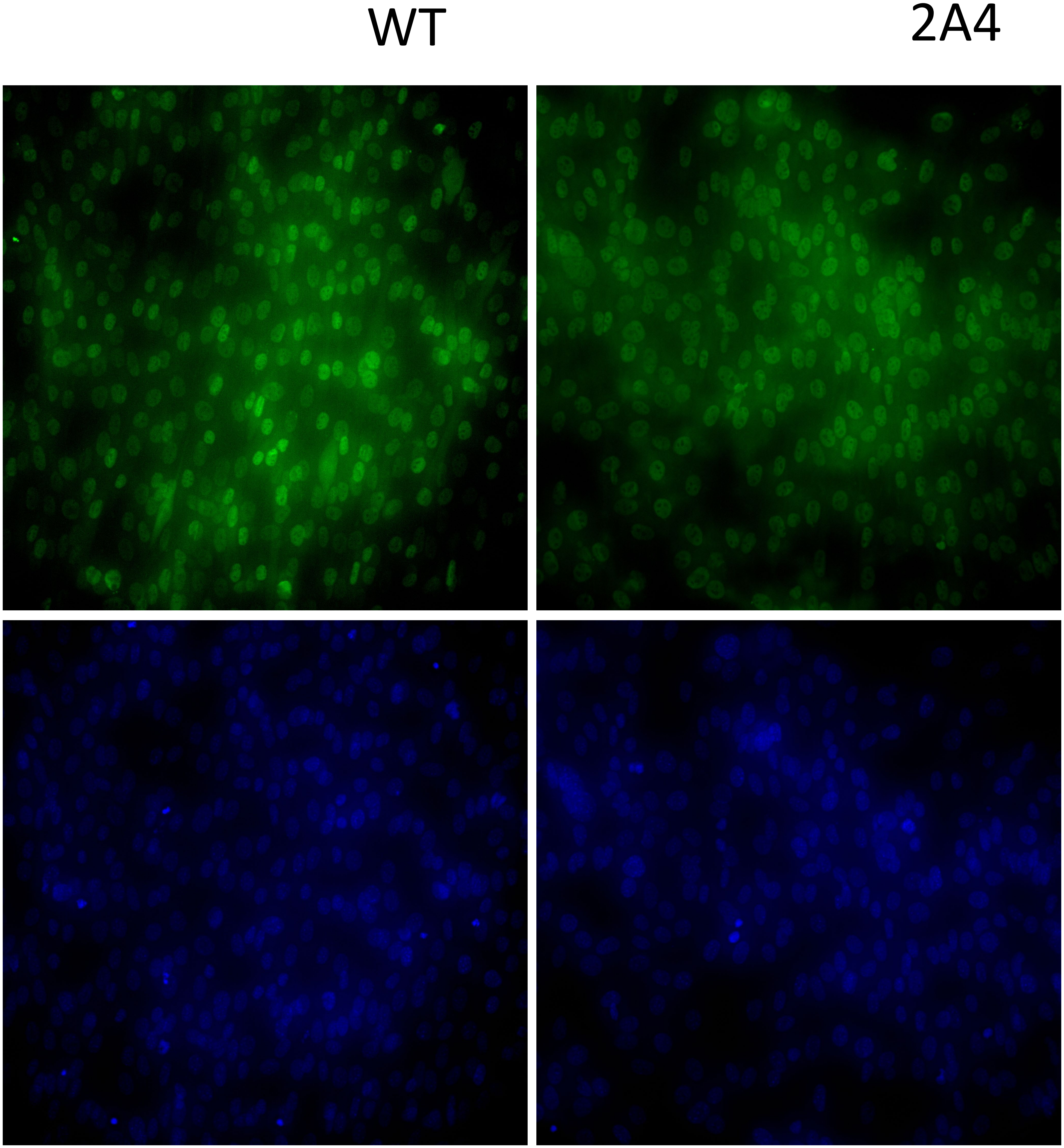

Day 4 MyoD

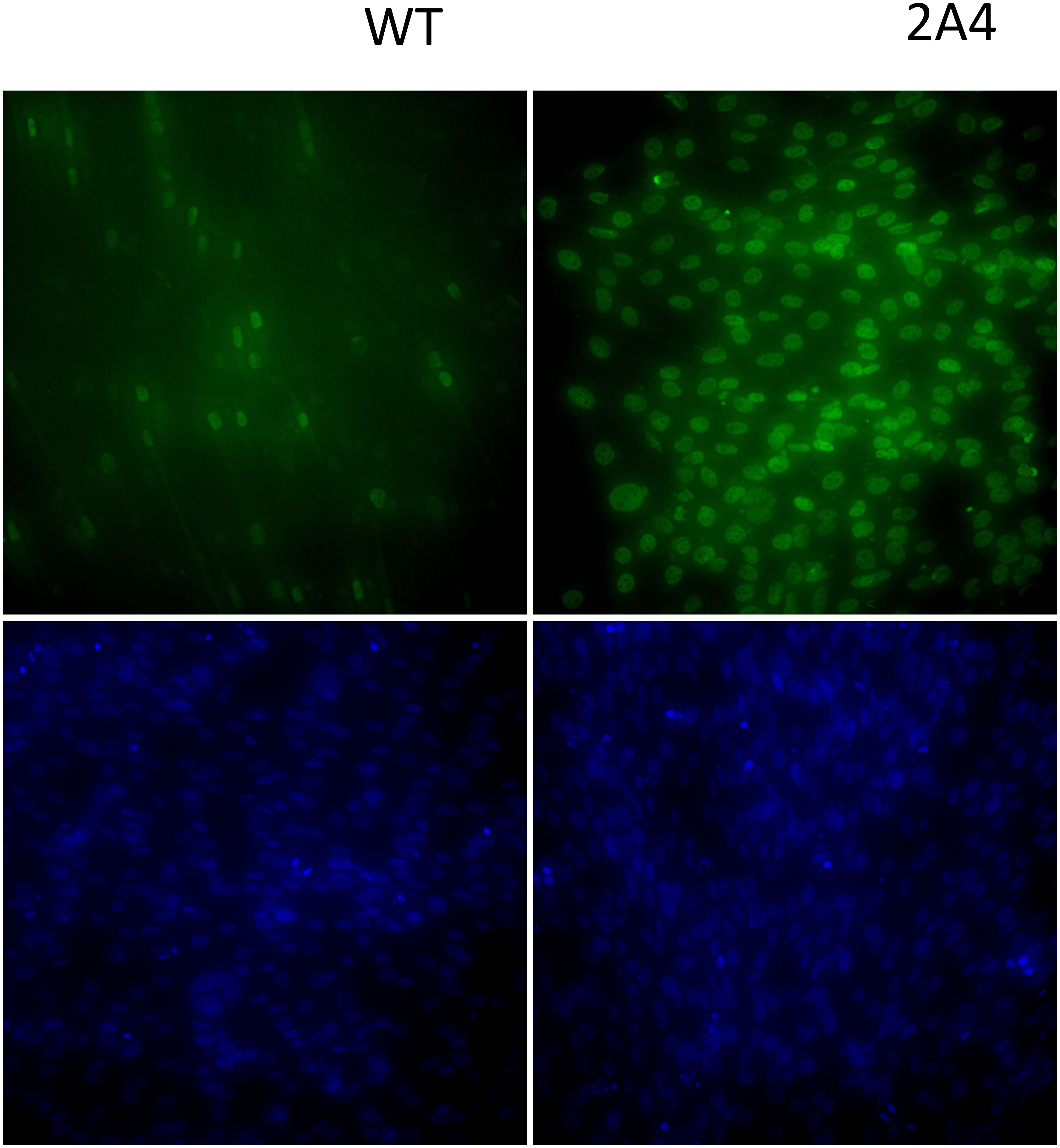

Day 0 Myogenin

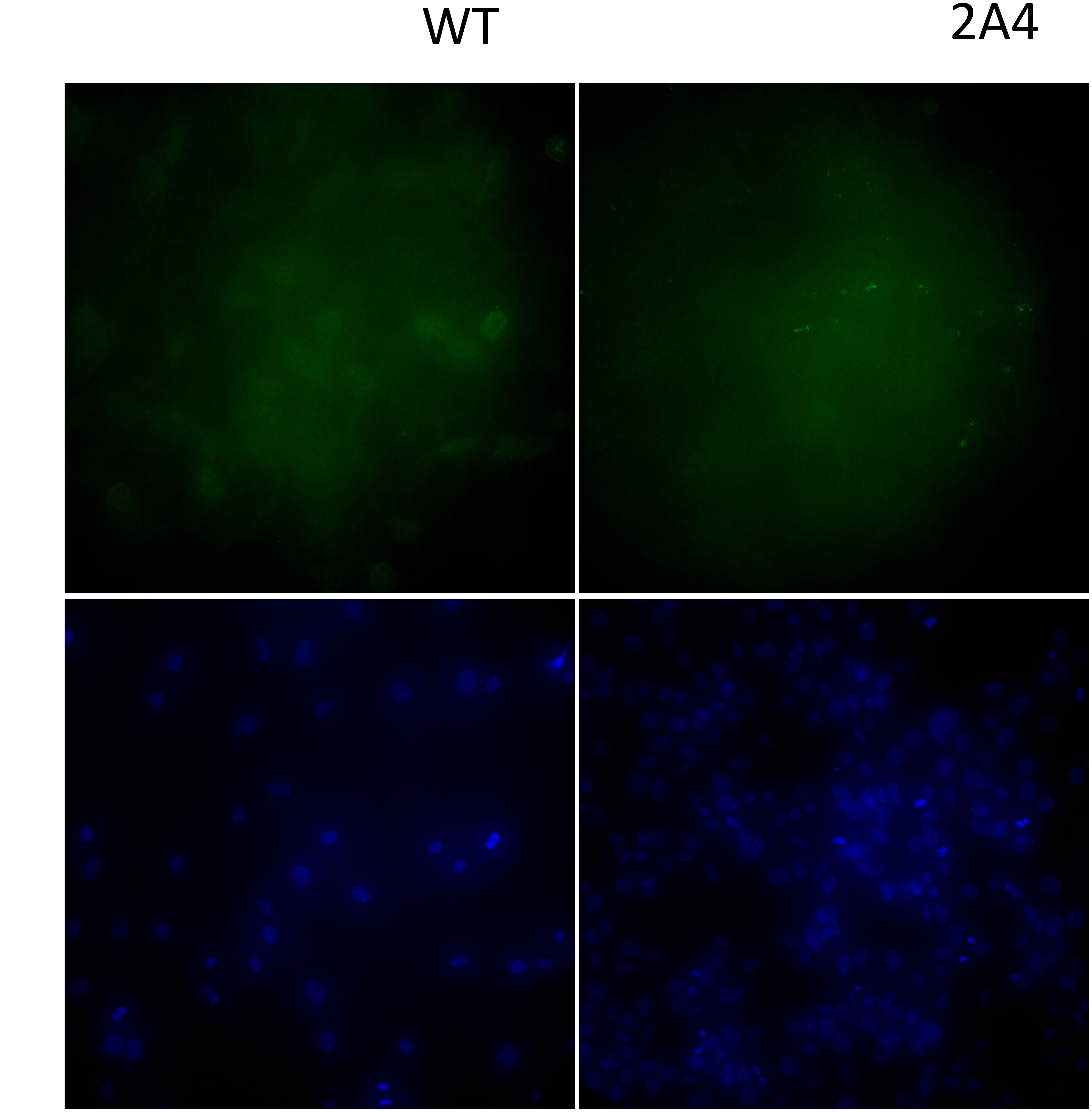

Day 1 Myogenin

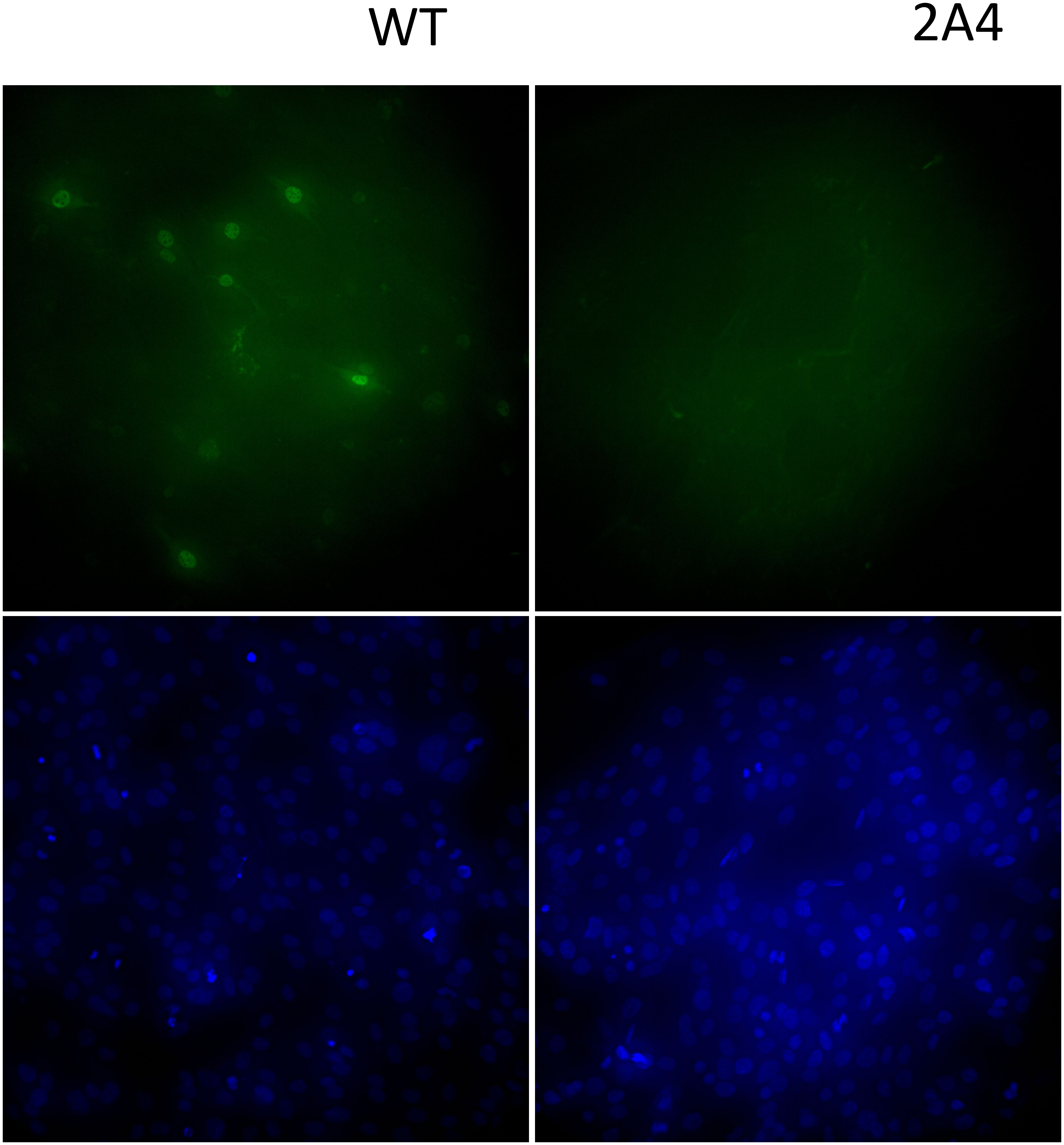

Day 2 Myogenin

WT 2A4

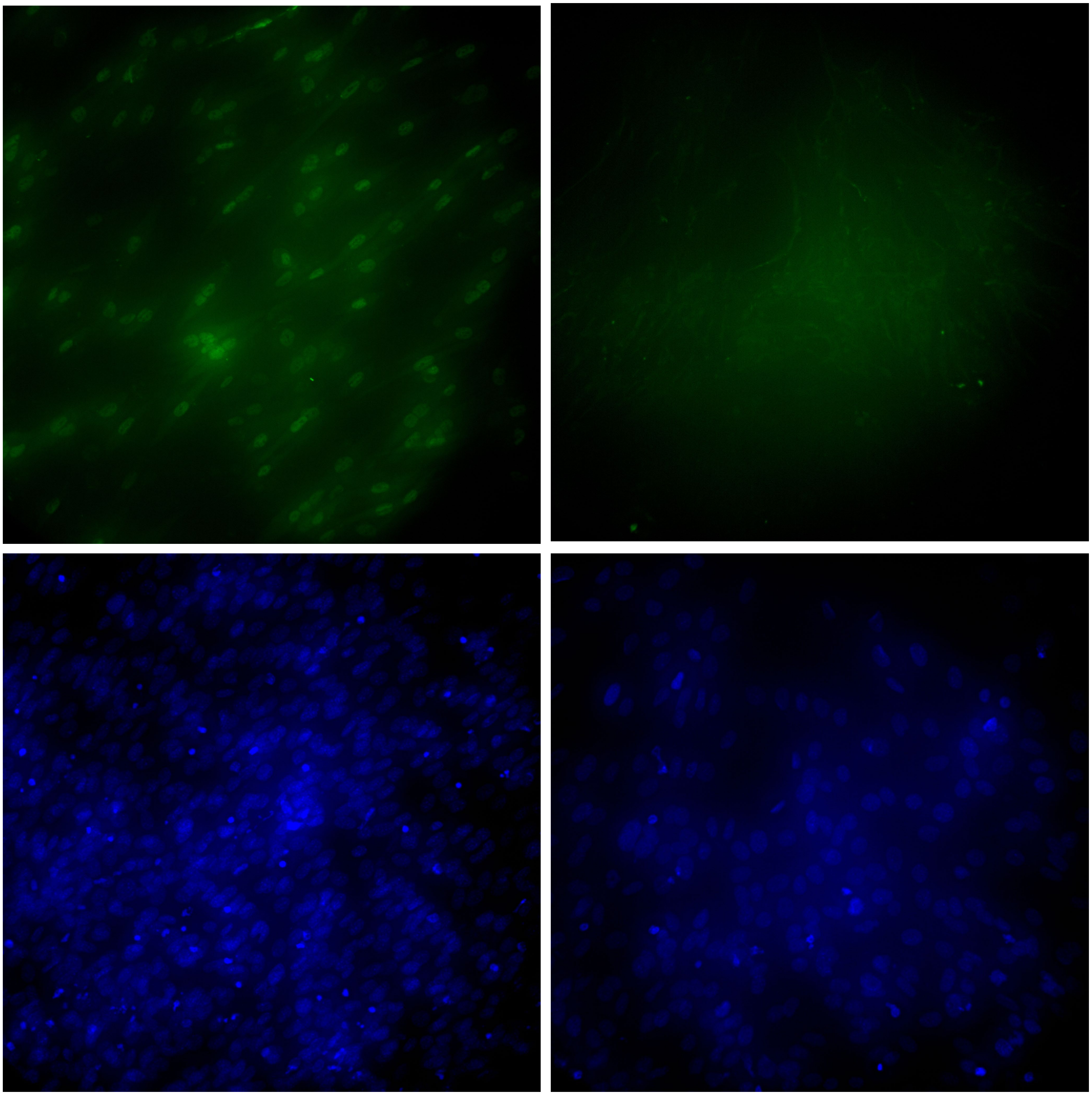

Day 3 Myogenin

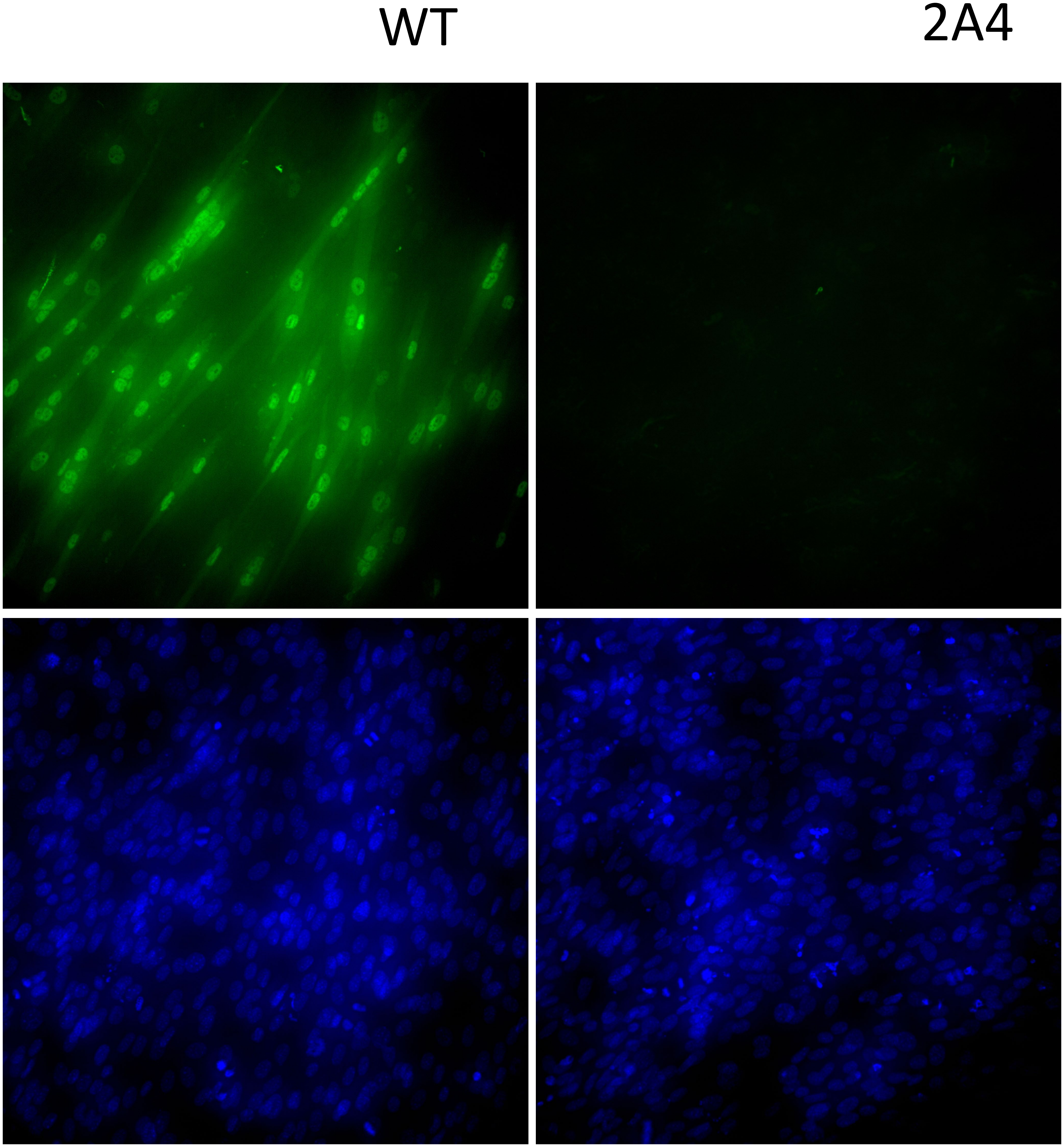

Day 4 myogenin

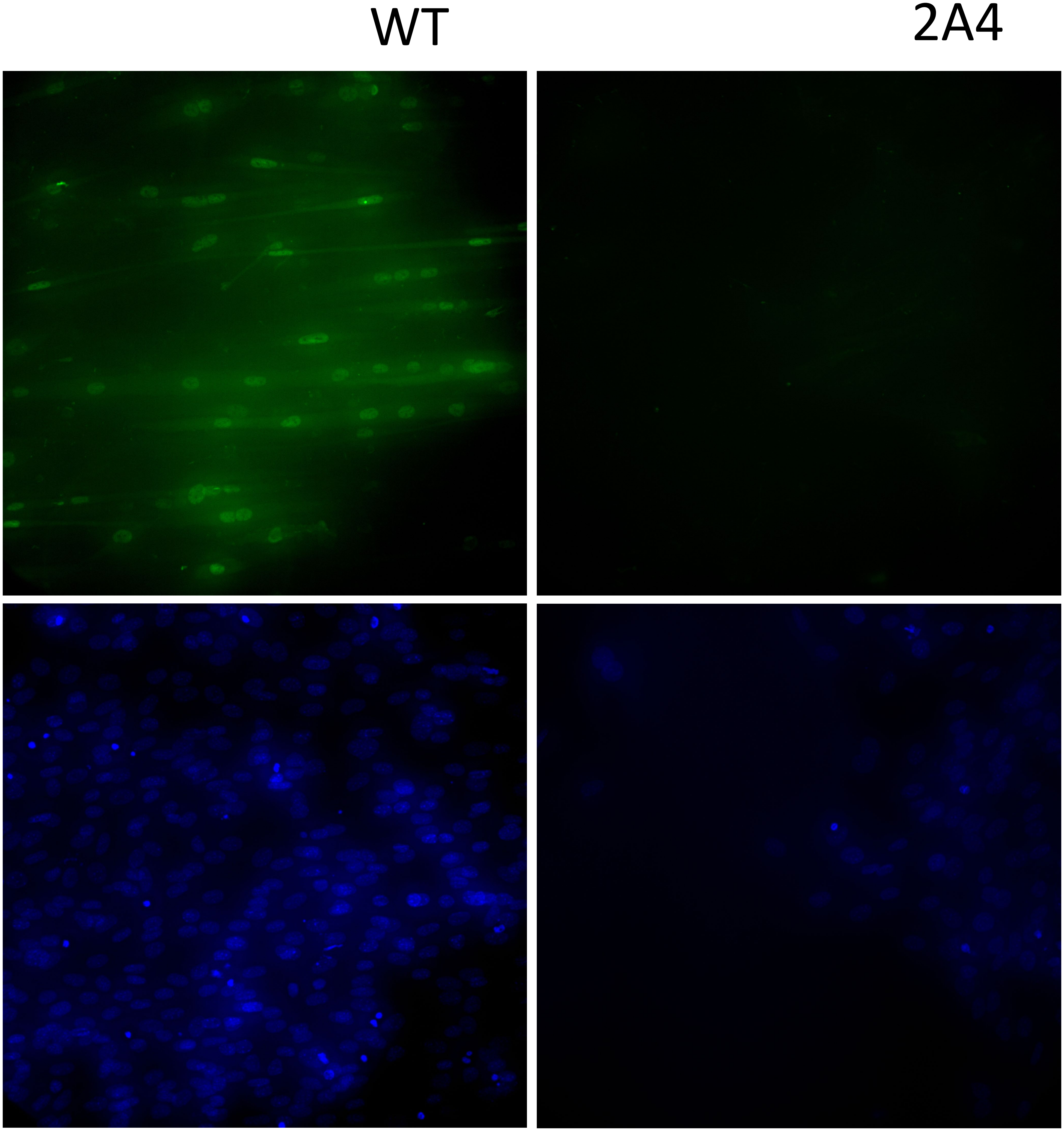

Day 0 MHC

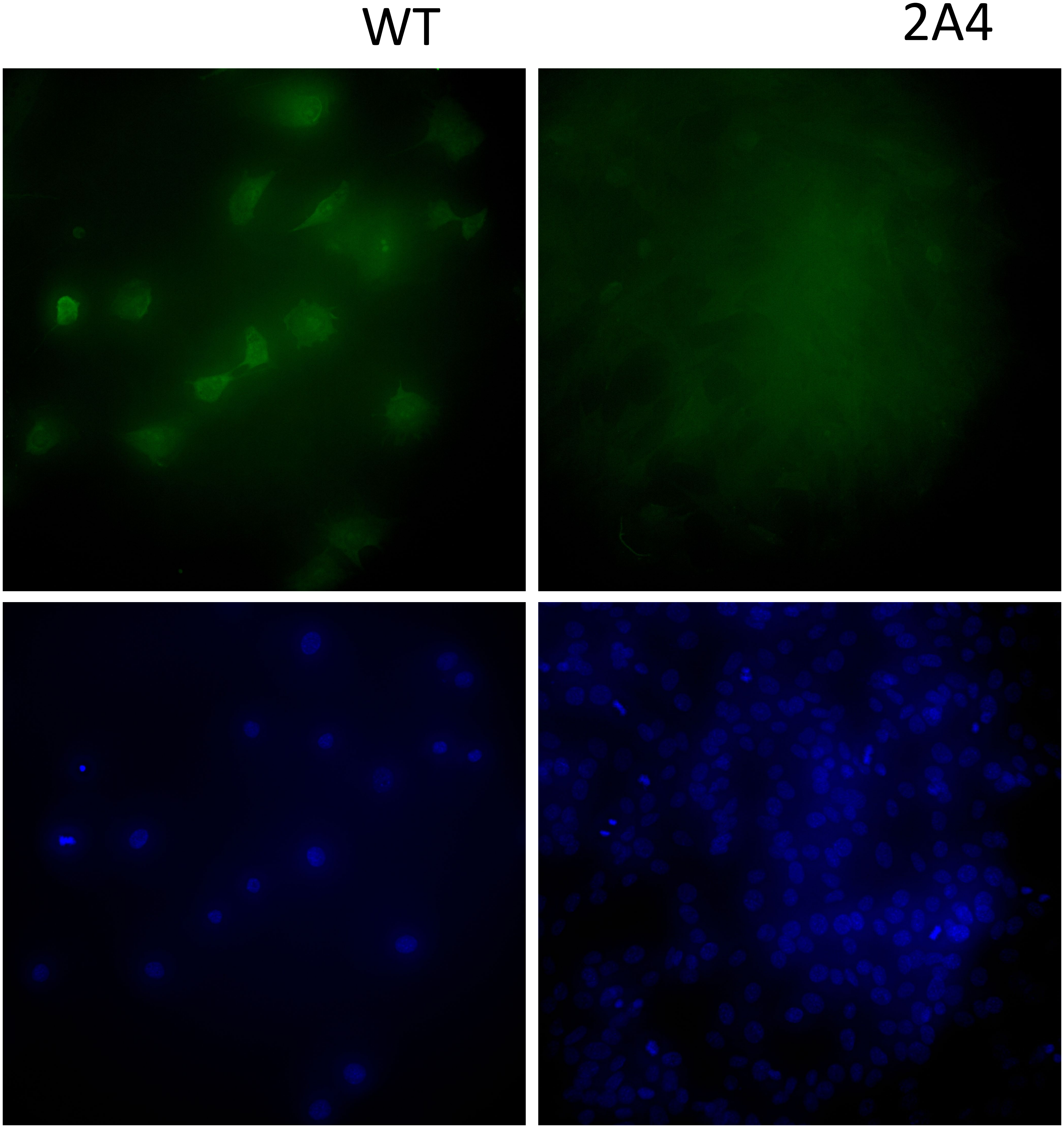

Day 1 MHC

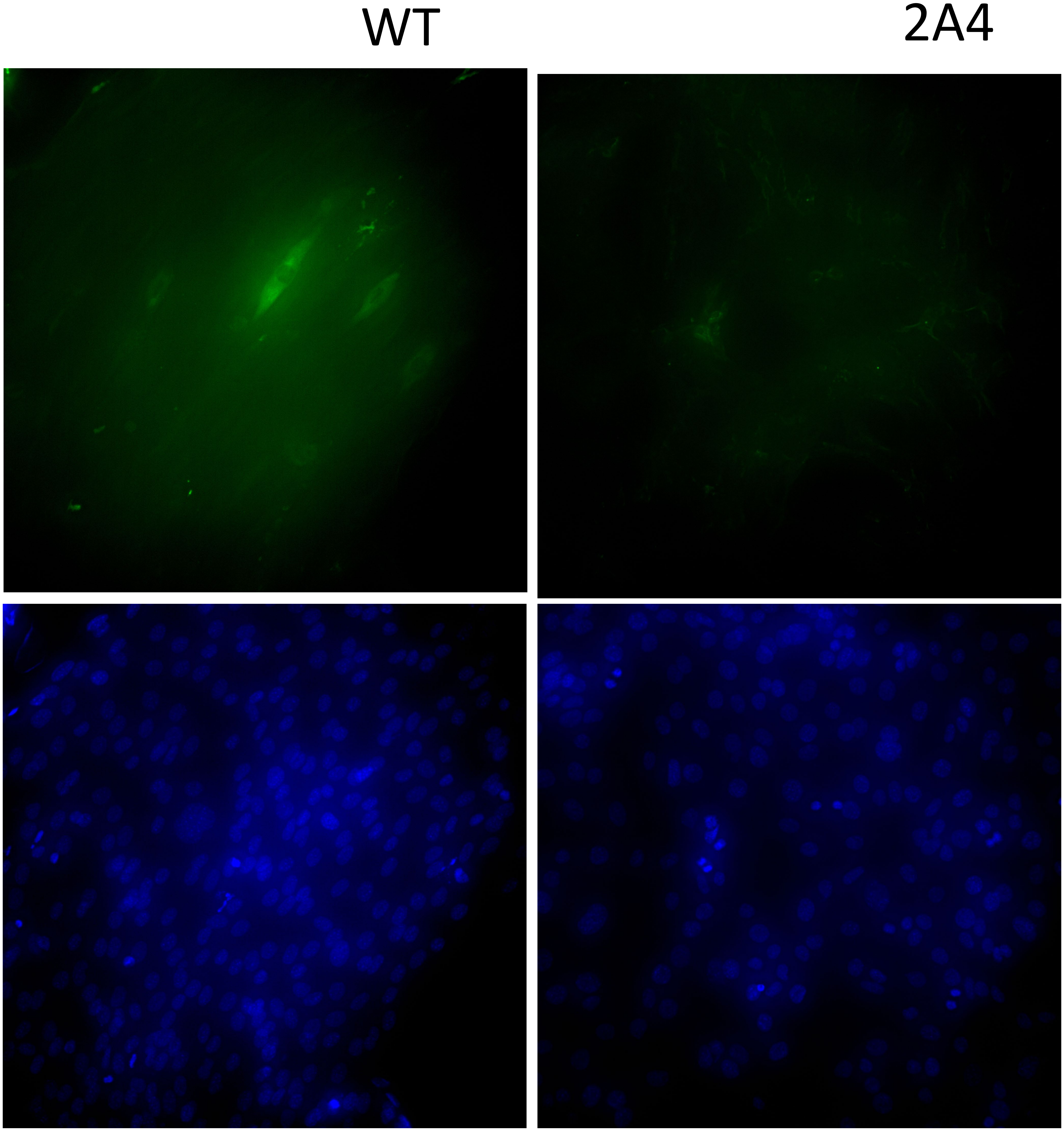

Day 2 MHC

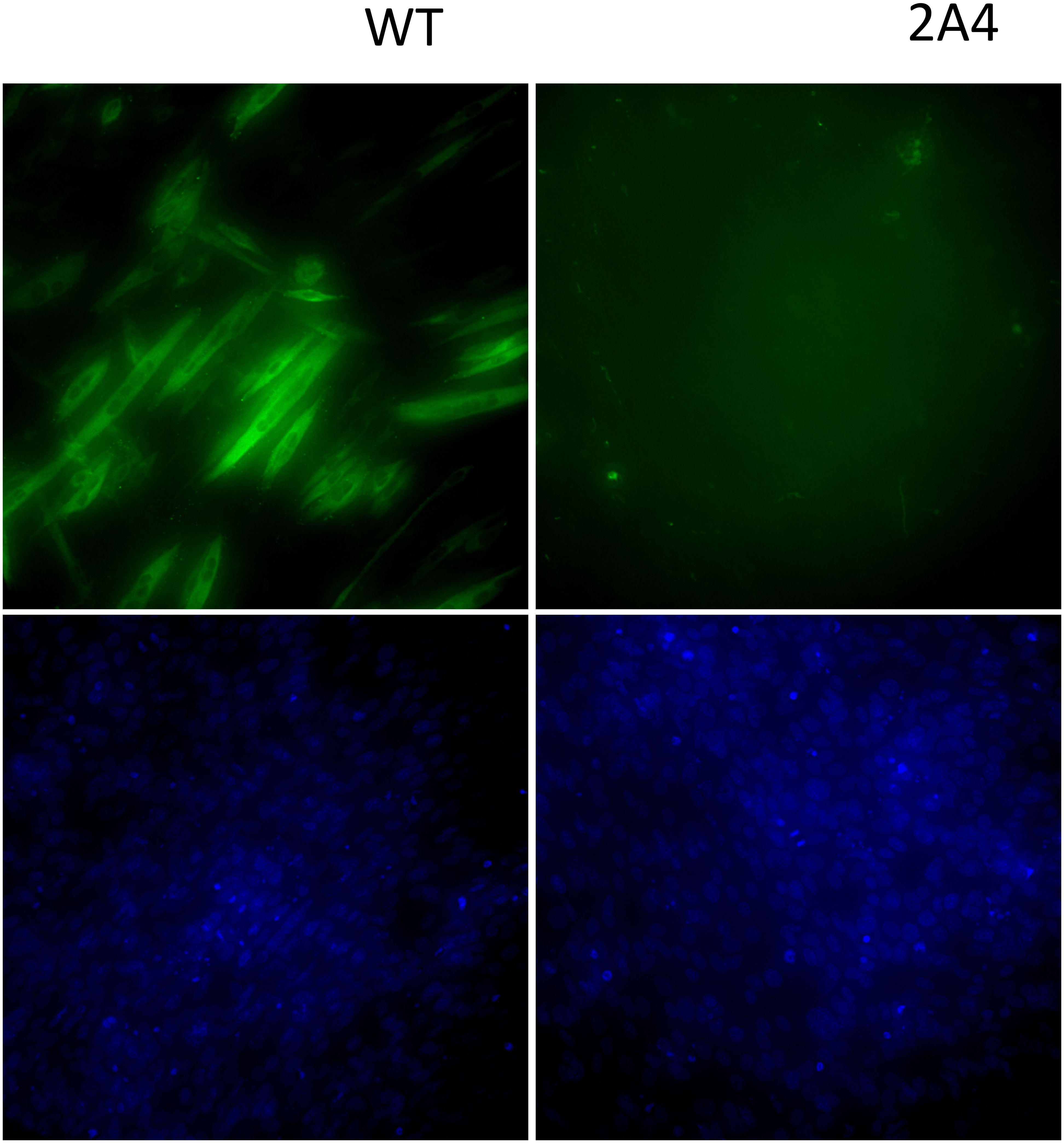

Day 3 MHC

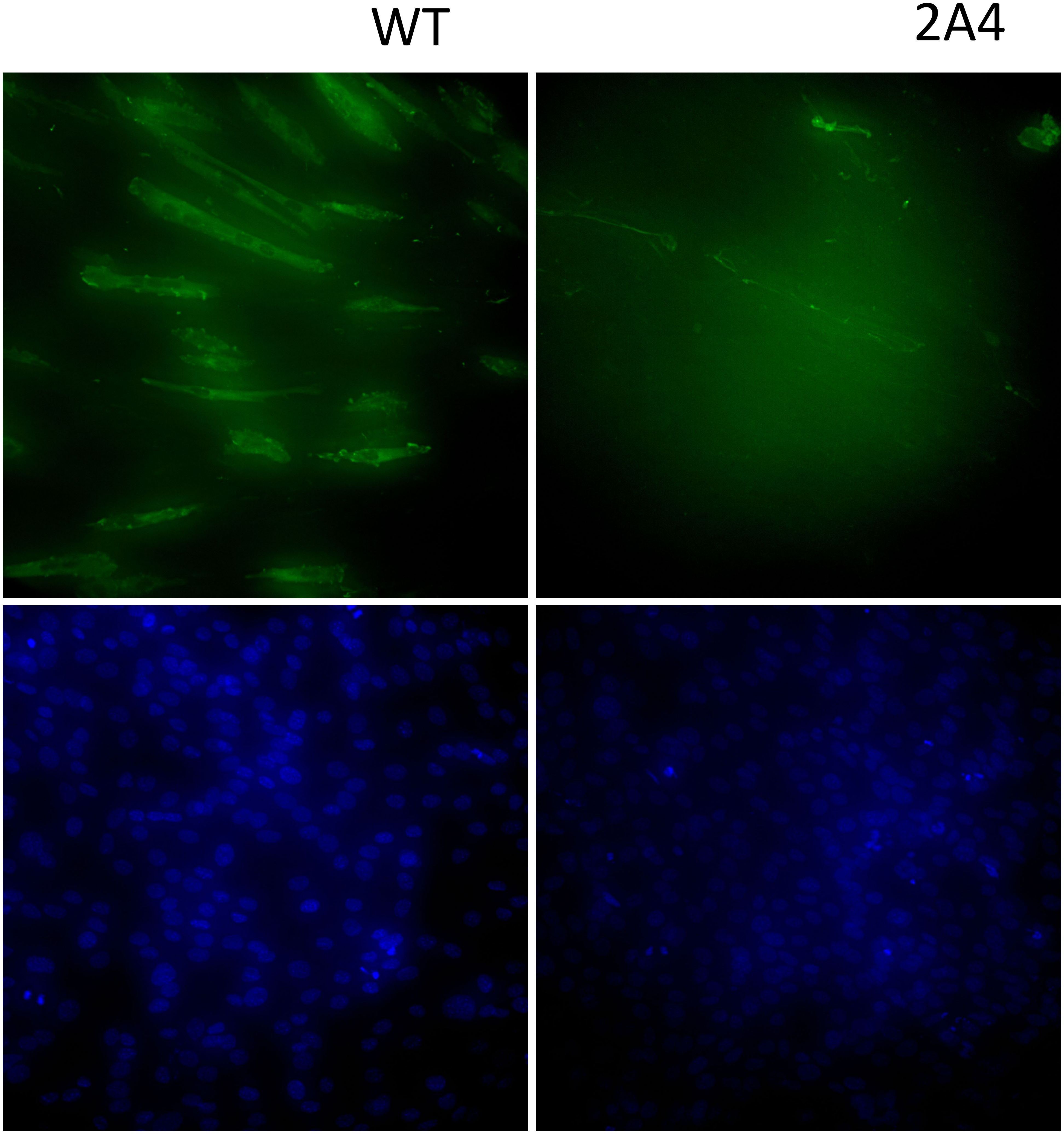

Day 4 MHC

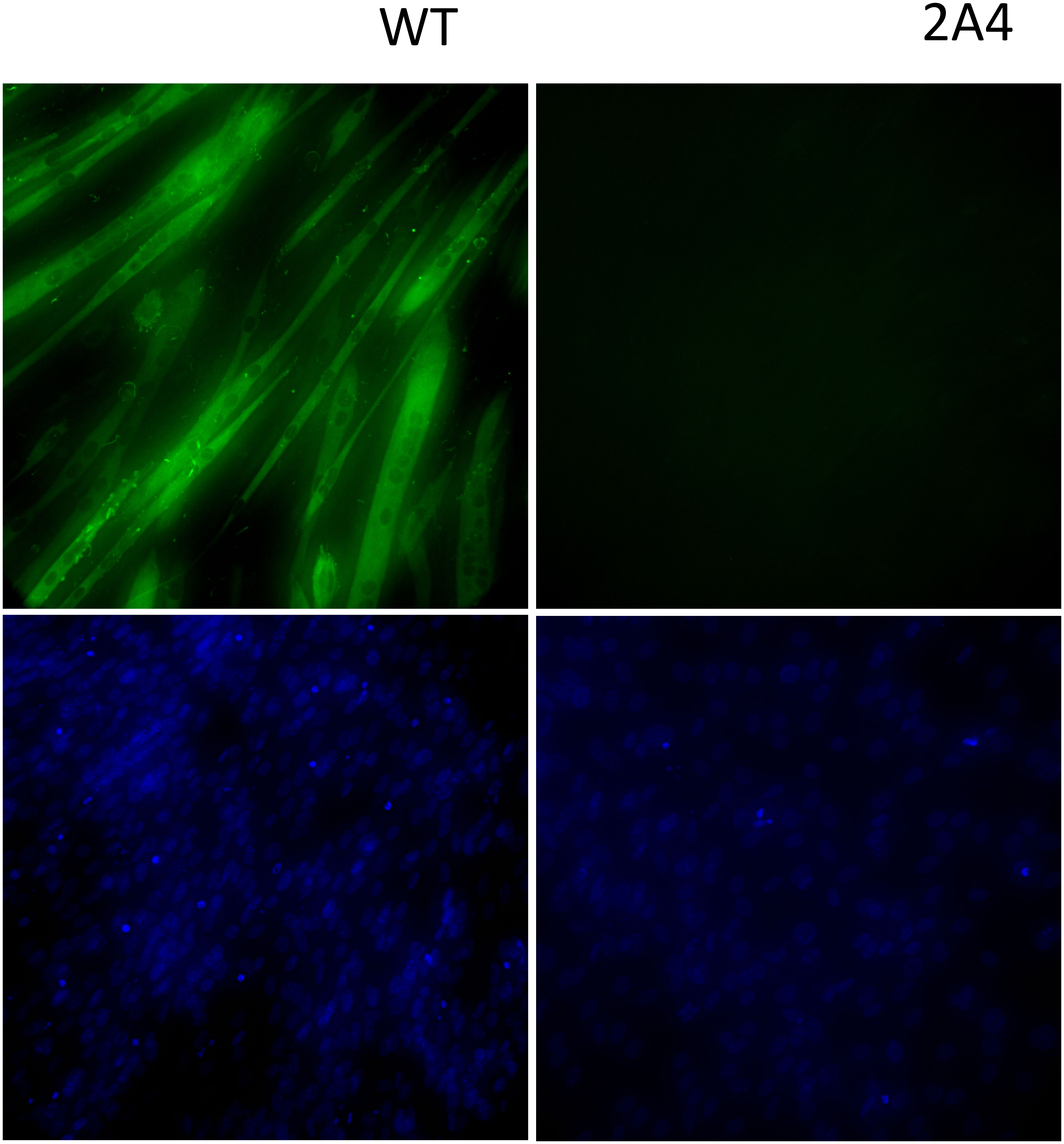

Day 0 Brightfield

WT

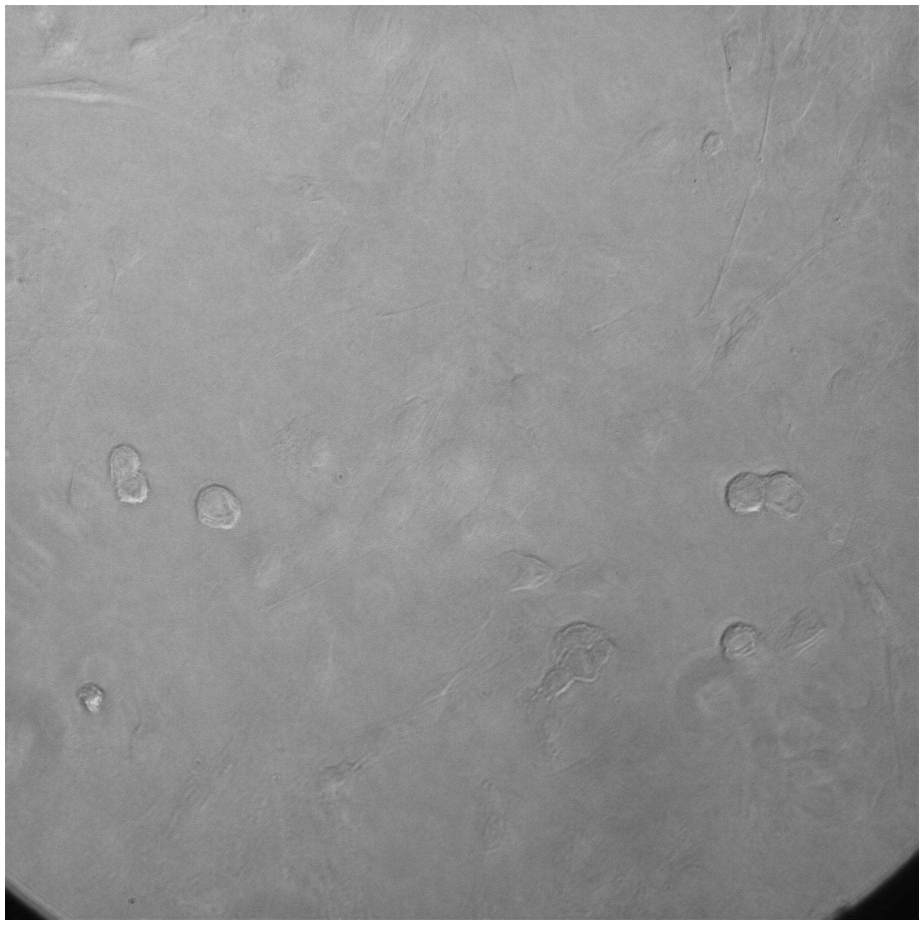

2A4

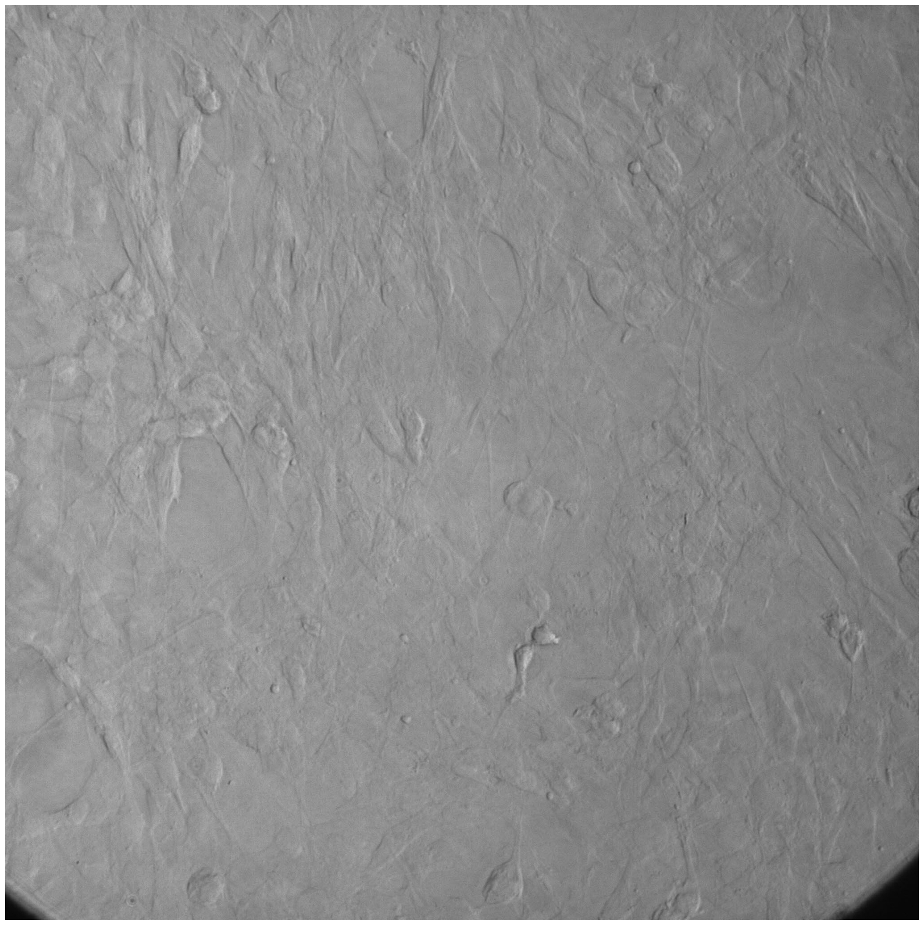

Day 1 Brightfield

WT

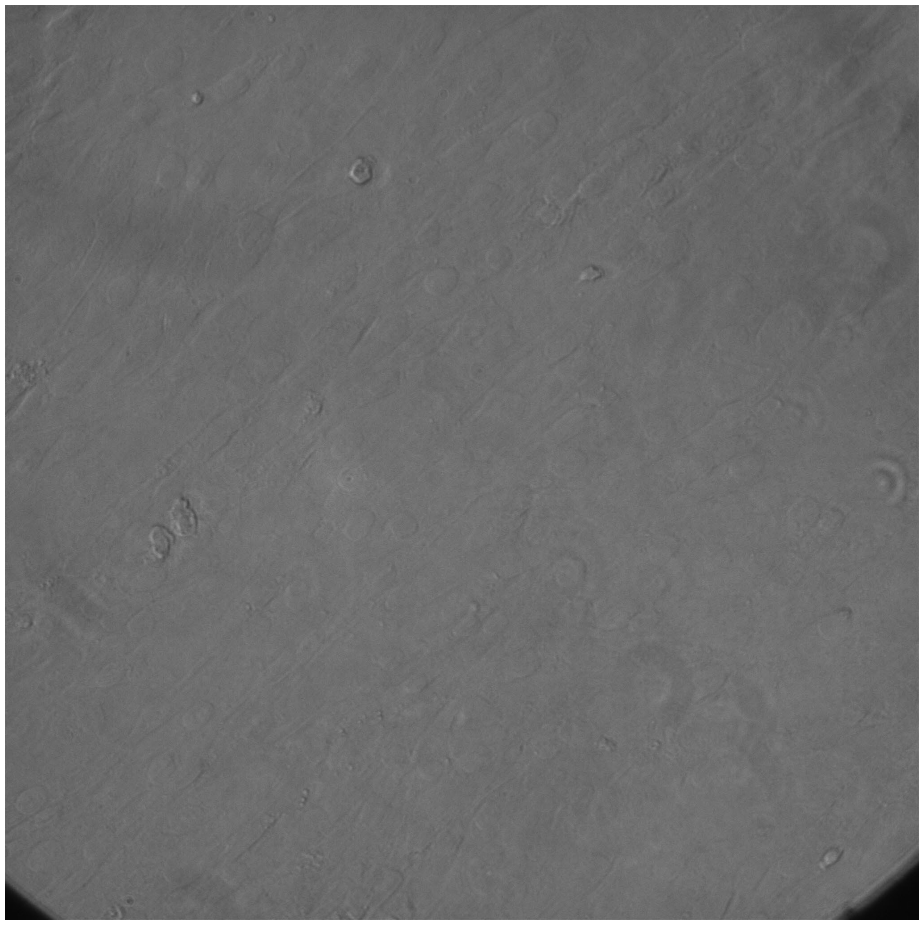

2A4

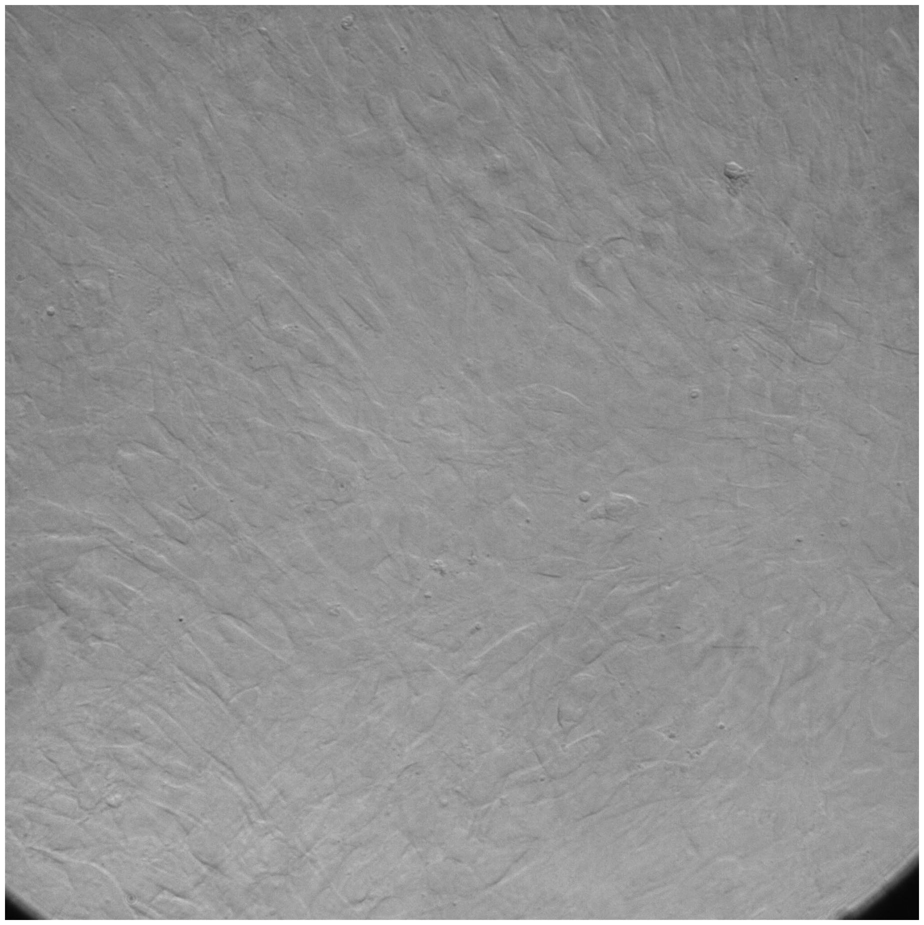

Day 2 Brightfield

WT

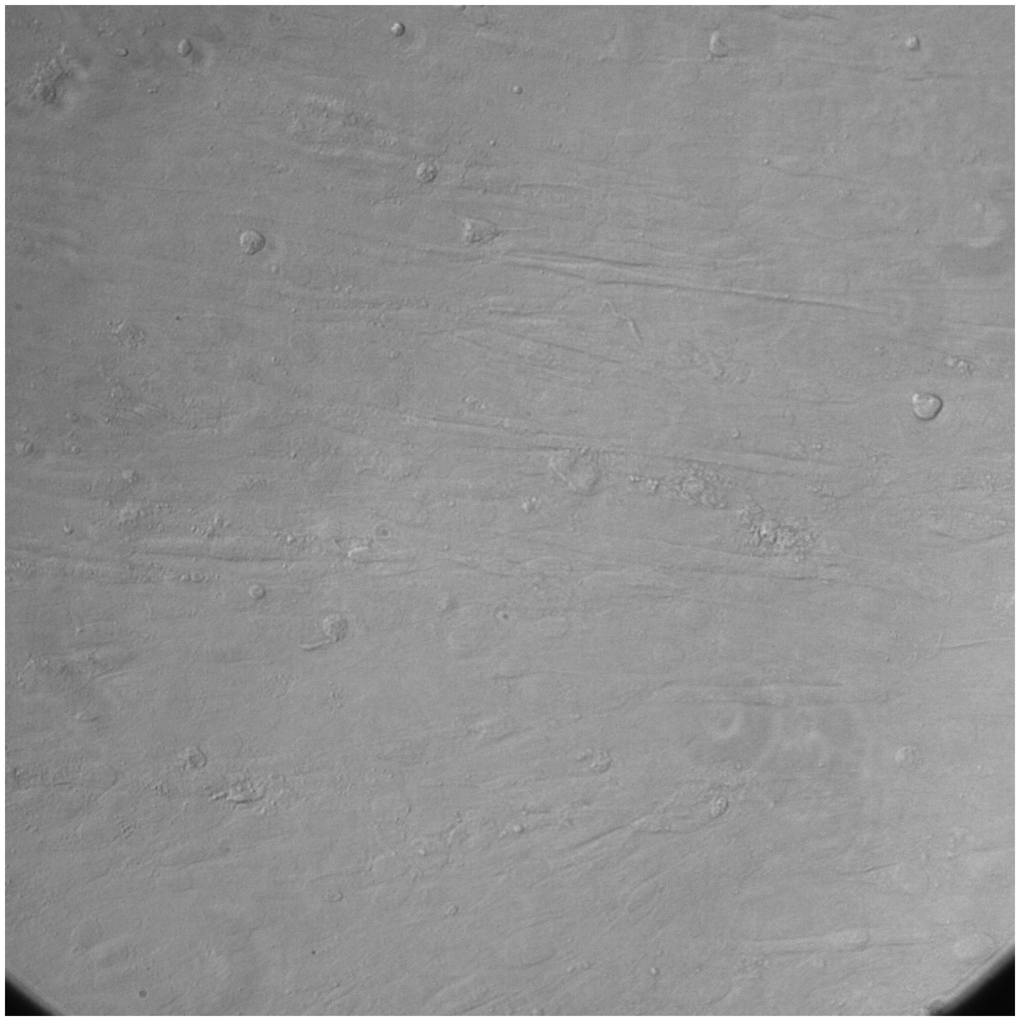

2A4

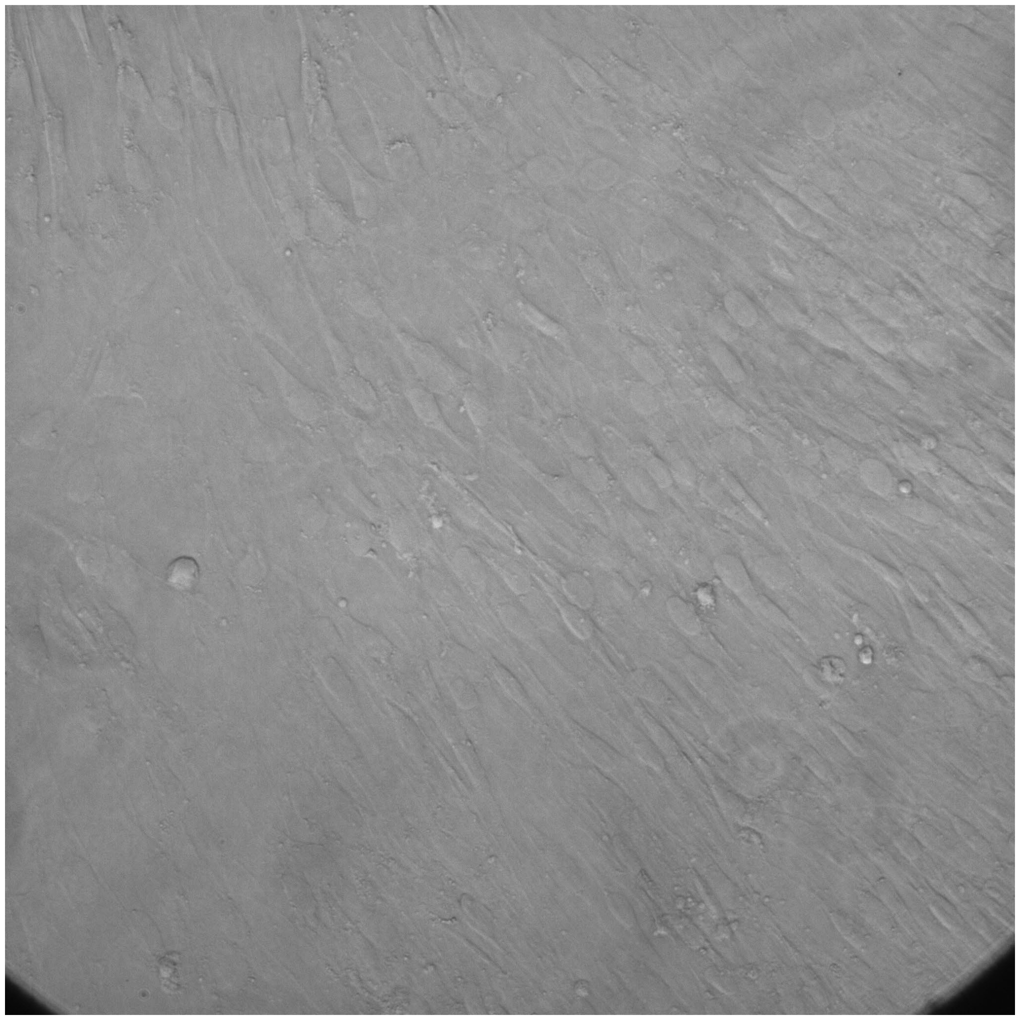

Day 3 Brightfield

WT

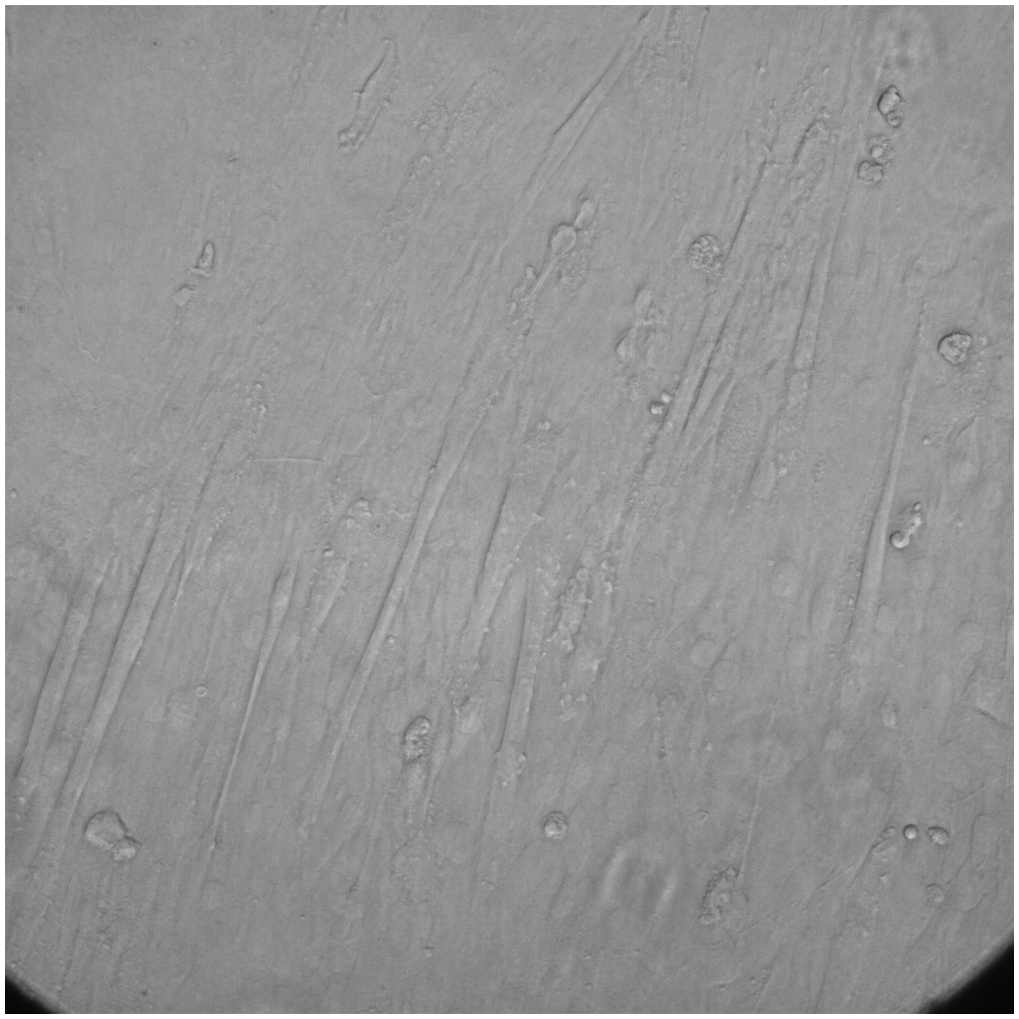

2A4

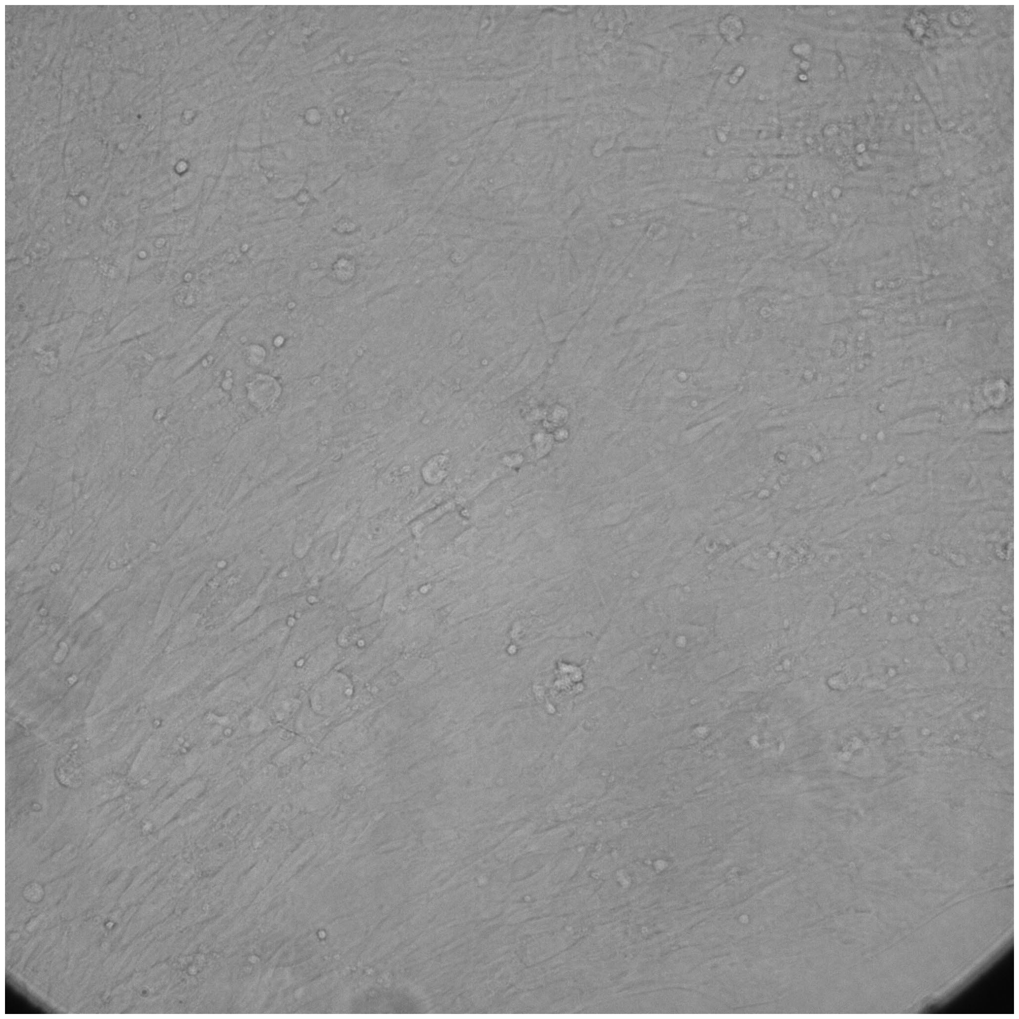

Day 4 Brightfield

WT

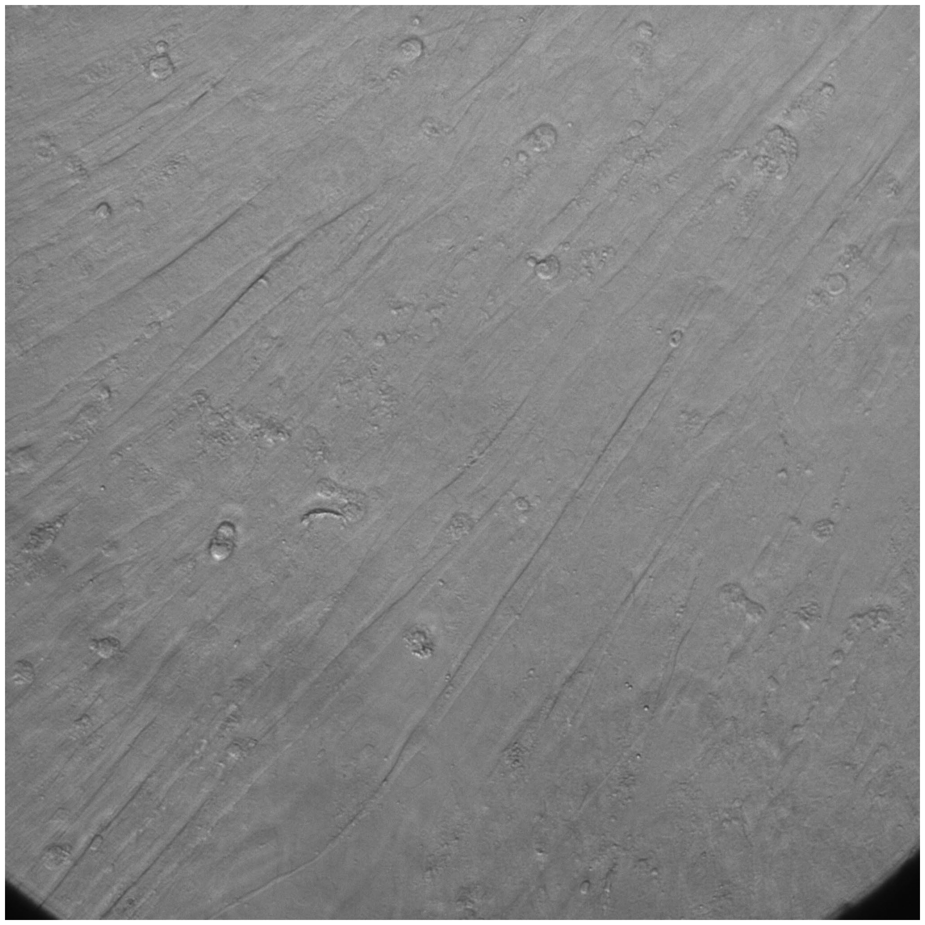

2A4

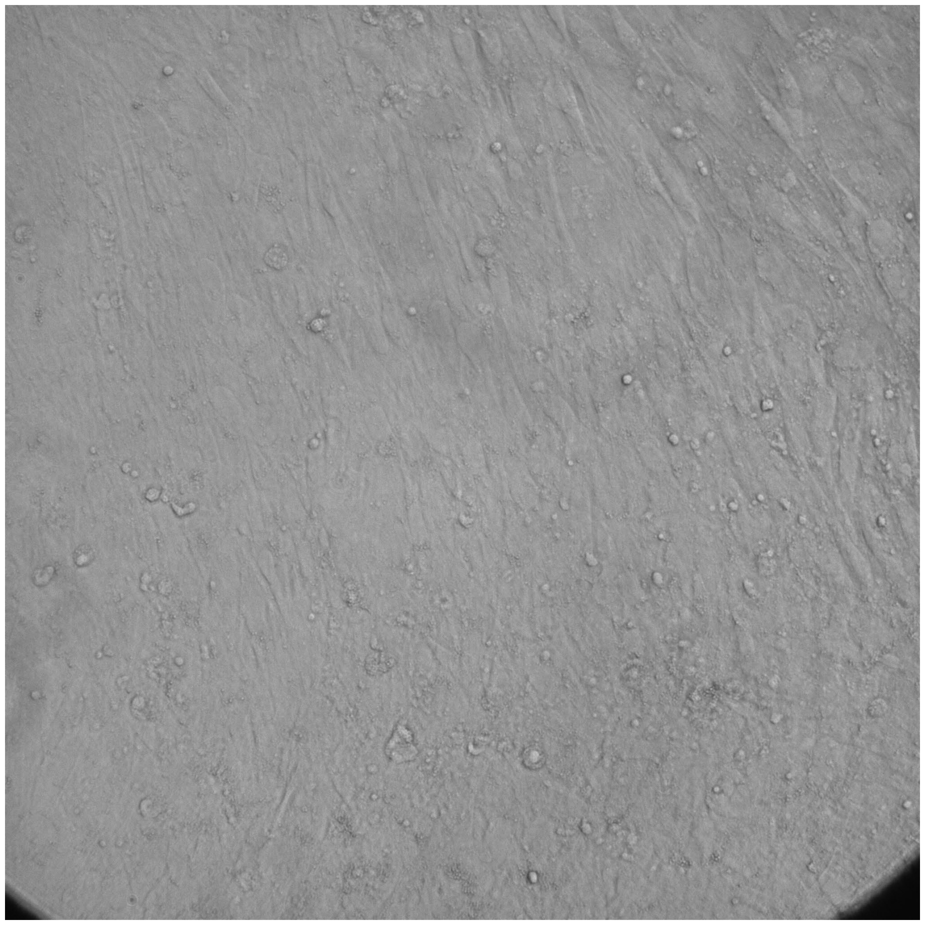

Supplement: S3 Dataset — Representative immunofluorescence images acquired for various myogenic factors and muscle proteins on days 0–4 of differentiation for wild type (WT) and CALM1 M109Q (2A4) C2C12 cells. (PDF) [file pone.0239047.s004.pdf]
